# Polycomb Repressive Complex 2 Modulation through the Development of EZH2–EED Interaction Inhibitors and EED Binders

**DOI:** 10.1021/acs.jmedchem.1c00226

**Published:** 2021-08-05

**Authors:** Stefano Tomassi, Annalisa Romanelli, Clemens Zwergel, Sergio Valente, Antonello Mai

**Affiliations:** † Department of Pharmacy, University of Naples “Federico II”, Via D. Montesano 49, 80131 Naples, Italy; ‡ Department of Chemistry and Technology of Drugs, “Sapienza” University of Rome, P.le A. Moro 5, 00185 Rome, Italy

## Abstract

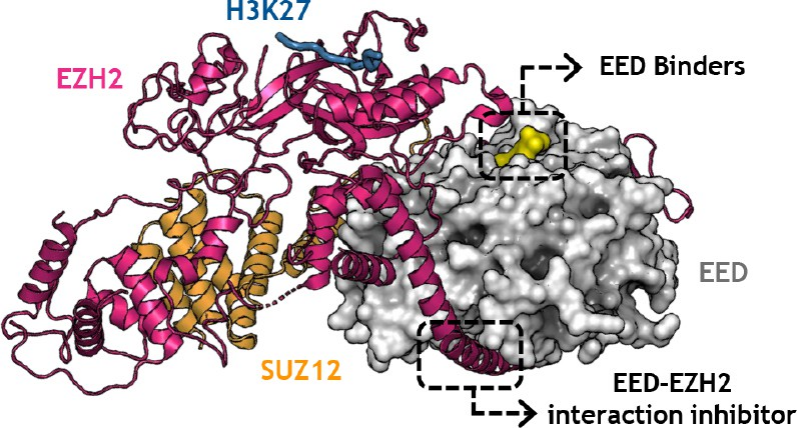

Epigenetics is nowadays a well-accepted area of research. In the last years, tremendous progress was made regarding molecules targeting EZH2, directly or indirectly. Recently tazemetostat hit the market after FDA-approval for the treatment of lymphoma. However, the impairment of EZH2 activity by orthosteric intervention has proven to be effective only in a limited subset of cancers. Considering the multiproteic nature of the PRC2 complex and the marked dependence of EZH2 functions on the other core subunits such as EED, in recent years, a new targeting approach ascended to prominence. The possibility to cripple the function of the PRC2 complex by interfering with its multimeric integrity fueled the interest in developing EZH2–EED protein–protein interaction and EED inhibitors as indirect modulators of PRC2-dependent methyltransferase activity. In this Perspective, we aim to summarize the latest findings regarding the development and the biological activity of these emerging classes of PRC2 modulators from a medicinal chemist’s viewpoint.

## 1 Introduction

The polycomb repressive complex 2 (PRC2) is part of the epigenetic machinery and belongs to the polycomb-group (PcG) of proteins that play a central role in how distinct expression patterns are positioned, maintained, and inherited in specialized cells of multicellular organisms.^1^ Due to its *S*-adenosyl-l-methionine (SAM)-dependent methyltransferase activity, the PRC2 was characterized as an epigenetic *writer* targeting the lysine 27 of histone H3 (H3K27) tail.^2^ However, the increasing understanding of how this post-translational modification (PTM) is directed or impeded on specific genomic sites by some of the PRC2 components has shed light on its additional *reading* functions.^3,4^ As a post-translational modifier, the PRC2 is able to catalyze with different propensity the mono- (me1), di- (me2), and trimethylation (me3) of H3K27;^5,6^ nonetheless, it can also target lysines from noncanonical substrates including JARID2, STAT3, and RARa.^7−9^ The H3K27 methylated states encode distinct epigenetic meanings, and whereas the H3K27me1 is ordinarily found in actively transcribed *loci*, the H3K27me3 mark is associated with a heterochromatin transition and silencing of developmentally important genes.^10^ In this regard, intriguing findings spotlighted that PRC2 not only installs *de novo* H3K27me3 tags but rather reinforces the established repressive state by fueling the so-called “spreading” of the H3K27me3 signal on already marked nucleosomes.^11,12^

Four interactors compose the human PRC2 complex: EZH2 or its homologue EZH1, which endows the complex with the histone methyltransferase (HMTase) capability, and three regulatory subunits (SUZ12, EED, and RbAp46/48).^5,6,13^ These noncatalytic members confer structural stability to the assembly and enhance its enzymatic activity and target recognition.^6,13−15^ However, the complex may also include facultative subunits, such as AEBP2, JARID2, the PCL1–3 proteins, PALI, and EPOP, which grant further regulating functions.^16−20^

EZH2 endows the PRC2 with catalytic competence through its structurally conserved C-terminal SET [Su(var)3–9, enhancer-of-zeste, and trithorax] domain.^21^ Although the SET domain is catalytically self-sufficient in other HMTase proteins, in the PRC2, it is unable to accomplish the methyl transfer by itself.^14,22,23^ When EZH2 is devoid of the core subunits EED and SUZ12, it is biologically unstable, and its SET domain switches to an inactive catalytic state that cripples the PRC2 methyltransferase capability and functions.^24,25^

EED carries out a further regulatory role by sensing the methylation status of already H3K27me3-tagged histones, exerting a positive allosteric control on EZH2 catalysis. Upon recognition of the H3K27me3 trimethylammonium motif in a shallow pocket of its β-propeller structure, EED prompts a structural reorganization of the SET domain, enabling a basal to stimulated catalysis shift and efficient H3K27me3 deposition by PRC2.^3,26^ Other trimethylated fragments were found to interact with EED *in vitro*, such as the stimulating JARID2-K116me3 (JARID2me3) and histone tail-derived peptides; albeit the latter do not establish any allosteric circuits.^7,26,27^ Furthermore, Muir and co-workers recently discovered that unmethylated H3K36 can increase the EZH2 activity through an ancillary sensing pocket, hence broadening the extent of this kind of feedback loop.^4^ Therefore, EZH2 may pass through distinct catalytic stages according to the PRC2 architecture and the chromatin context: an autoinhibited configuration when it lacks the EED and SUZ12 pair,^23^ a basal active state within the PRC2 ternary assembly,^6^ and an H3K27me3-dependent stimulated state.^26,27^

Dysregulation of the PRC2–H3K27me3 axis is linked to different diseases, including several cancers,^28,29^ viral infections,^30,31^ the Weaver syndrome,^32,33^ and inflammation processes.^34^ In cancer, EZH2 is generally considered oncogenic and, together with the other core subunits, was found to be overexpressed and correlated with poor prognosis in a multitude of solid tumors.^35−37^ Genome sequence analysis also revealed that gain-of-function mutations of EZH2 (Y641, A677,^38^ and A687) are abundant among varied forms of lymphomas.^38−42^ PRC2 components may also act as tumor suppressors and undergo loss-of-function or missense mutations in T-cell acute lymphoblastic leukemia and myelodysplastic syndromes.^36,43,44^

**Figure 1:**
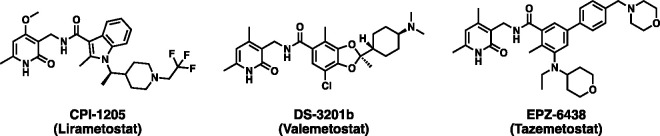
Catalytic EZH2 inhibitors under phase I/II clinical studies.

Taken together, these pieces of evidence endorsed the PRC2 as a top-ranked target for cancer treatment, and in this respect, extensive med-chem campaigns were launched to develop small molecular entities tackling its functions. As catalytic subunit, EZH2 was considered the ideal target to shut down abnormal PRC2 activities directly, and several SAM-competitive inhibitors, sharing a pharmacophoric 2-pyridone scaffold, have been developed with the time. Such inhibitors demonstrated HMTase-dependent activities both *in vitro* and *in vivo*, and some of them (CPI-1205, SHR2554, DS-3201b, CPI-0209) are presently undergoing phase I/II clinical investigations for solid or hematological tumors, as deeply discussed in recent reviews.^45−47^ Notably, 2020 saw the FDA-approval of the first SAM-competitive anti-EZH2 drug, EPZ-6438 or tazemetostat (Tazverik), which has been authorized for the care of locally advanced epithelioid sarcoma and follicular lymphoma.^48−50^ An overview of the EZH2 inhibitors currently in clinical trials is reported in Figure 1.

More and more EZH2 inhibitor drugs are awaited in the near future; however, such agents are not free of drawbacks: they have proven efficacy in a limited subset of cancers, mainly in Y641 or A677 mutant lymphoma cells, and their prolonged administration aroused different adaptive cancerous response mechanisms, such as activation of the insulin-like growth factor 1 receptor (IGF-1R), MEK, and phosphoinositide-3-kinase (PI3K) pathways, that ultimately restricted the overall clinical outcome.^51^

Considering the multiproteic nature of the PRC2 complex and the strict dependence of EZH2 activity on the scaffolding and regulating roles of EED, in recent years, alternative inactivating strategies gained attention. On one side, the possibility to cripple the PRC2 complex functions by interfering with the intimate protein–protein interaction (PPI) between EZH2 and EED fueled the development of different chemotypes as EZH2–EED protein–protein interaction (PPI) inhibitors. These chemical agents exert a methyltransferase inhibitory activity on PRC2 by impeding the scaffolding role of EED on EZH2-SET domain correct folding, as discussed in the following related paragraph. On the other hand, as anticipated in a seminal viewpoint,^52^ a high throughput screening by Novartis demonstrated the “druggability” of the H3K27me3-recognizing cavity of EED as a means to allosterically inhibit EZH2 catalysis. The ensuing hit-optimization process has led to EED binders with improved physicochemical and biological properties *in vivo*, ultimately providing a clinical candidate (MAK683).^53,54^

In this Perspective, we aim to present these emerging classes of PRC2 modulators and to summarize the latest findings regarding their development and biological activity from a medicinal chemist’s viewpoint.

## 2 The PRC2 Core Complex Architecture and Its Transition between Catalytic States

**Figure 2:**
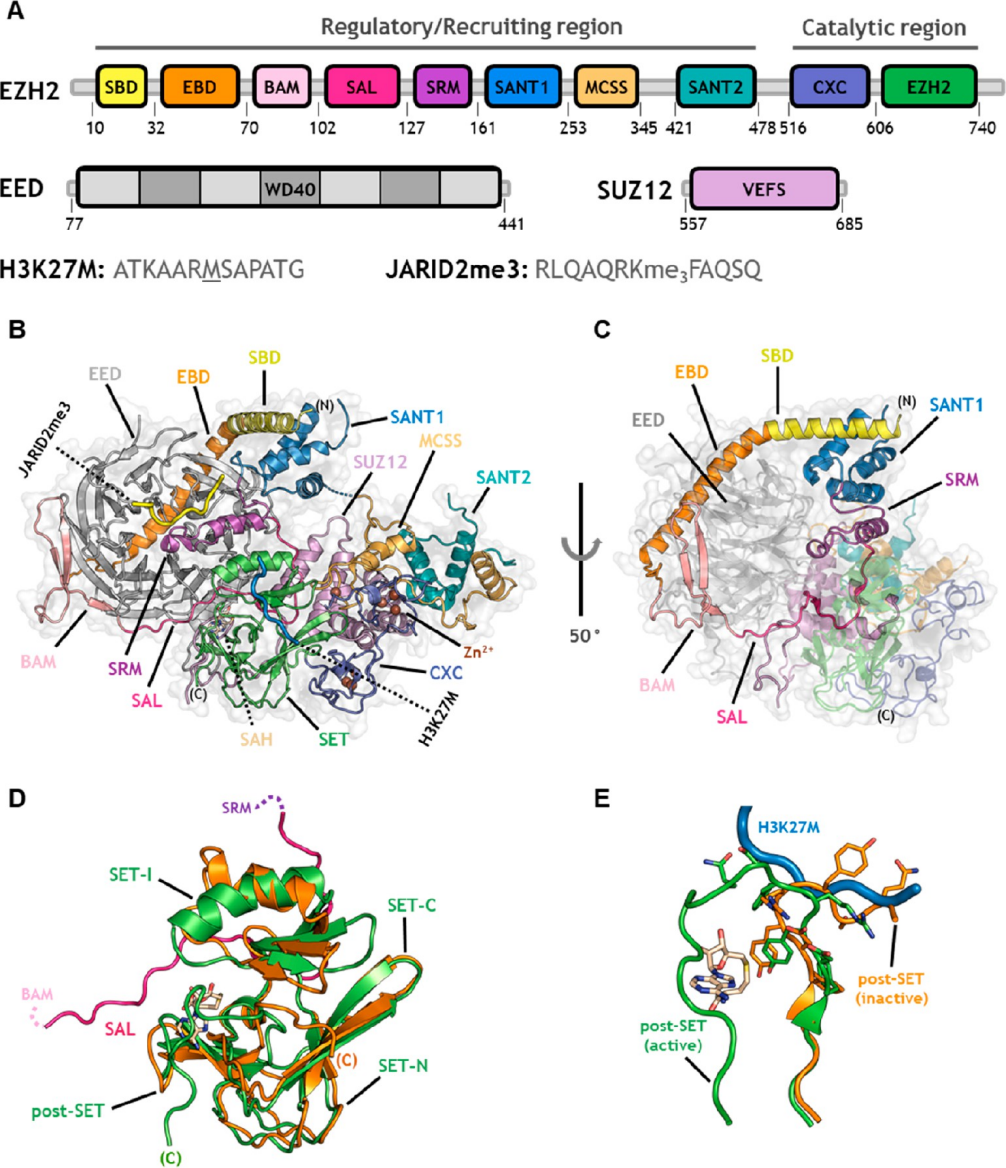
Overall architecture of the human PRC2 core complex and stabilization of its basal catalytic state. (A) Schematic representation of the PRC2 complex subunits. The colors of the EZH2 domains are in accordance with their representation in panels B and C. H3K27M and JARID2me3 peptide sequences are in one-letter code. Mutant methionine is underscored. (B) Structure of the catalytically active PRC2 core complex in cartoon and semitransparent surface representations (PDB 5HYN). PRC2 subunits and EZH2 domains are labeled and highlighted in varied colors. H3K27M and JARID2me3 peptides are in blue and yellow cartoon, respectively. SAH is in stick representation. (C) Rotated view (*Y* = 50°) of the complex with the EZH2 domains, forming the belt-like structure, labeled and highlighted in colors. (D) Superimposition of the human EZH2 SET domain in isolated inactive (PDB 4MI5, orange) and catalytically active conformation (PDB 5HYN, green). (E) Superimposition of the post-SET subdomain from the inactive (orange) and active conformation (green), showing its outward torsion that clears the H3K27M (blue) binding cleft.

EZH2, EED, and SUZ12 subunits interact through extended molecular surfaces, and different X-ray cocrystal structures appear in the literature showing the molecular contacts engaged in the core assembly, the target recognition, the nucleosomal recruitment, and the catalytic pocket organization.^55−60^ The human PRC2 core complex (EED, EZH2, and SUZ12-VEFS domain) was cocrystallized with the histone-derived oncogenic H3K27M_21–33_ false-substrate (H3K27M) lying in the substrate-binding furrow and the JARID2_110–122_-K116me3 peptide (JARID2me3) bound in the EED central pore (Figure 2A,B).^59^ This assembly has a three-fold structure and is functionally divided into a regulatory portion (corresponding to EED and the N-terminal segments of EZH2), a catalytic region (encompassing the C-terminal domains of EZH2 (CXC and SET)), and a middle section (bridging the previous ones and including the C-terminal α-helical bundle of SUZ12 (SUZ12-VEFS) and the EZH2-SANT2/MCSS domains).

In this assembly, the regulatory-adaptor protein EED occupies a central location and arranges multiple contacts with the different EZH2 segments. EED is a WD40-repeat-containing protein distinguished by a seven-bladed β-propeller architecture with one blade formed by a four-stranded antiparallel β-sheet (WD40 motif).^61^ The radial arrangement of the WD40 motif outlines two different-sized surfaces (top and bottom), which serve as a scaffold for the belt-like arrangement of the EZH2 N-terminal region (residues 10–246) on EED (Figure 2C). On one side, an extended α-helical stretch of EZH2 (EBD or EED binding domain) sits within a sharp gorge defined by the loops connecting the β-propeller blades and spans the length of the EED bottom surface.^59−61^ On the other side, EZH2 assumes a more unstructured conformation and interacts with EED through two flexible segments, SAL (SET activation loop) and SRM (stimulation-responsive motif). This “embracing” configuration is additionally secured by contacts given by the SBD (SANT1-binding domain) that intramolecularly docks into the distal SANT1 α-helix bundle and acts as a molecular clasp. EZH2 proceeds as a string of mainly α-helical domains (SANT1, MCSS, SANT2) with further stabilization roles and cementing SUZ12-VEFS domain to the core structure.^59,60^ Finally, the C-terminal CXC (or pre-SET) and SET domains compose the catalytic region of EZH2 and locate in close proximity to the SAL and SRM domains. Given the relevance as catalytic portion and the differential conformational plasticity, SET is additionally split into four subdomains, namely, SET-C, SET-I, SET-N, and post-SET, which delineate the substrate and cofactor binding sites.^62^

The described EED/SUZ12-dependent organization is essential to ensure the catalytic ability of the otherwise autoinhibited EZH2 subunit. Crystallographic alignments of the EZH2, alone or complexed with the core members, revealed consistent structural rearrangements at both the substrate and the cofactor binding sites, which become misfolded or self-seized by distinct SET portions.^22,59,60,63^ Indeed, in isolated EZH2, the SET-I and the post-SET subdomains undergo body rotations that bring them to fit within the histone binding cleft (orange structures in Figure 2D,E). This inactivating configuration demonstrates the critical role of EED as an adaptor protein since the organization around its β-propeller positions the SAL segment to stretch out at the border between EED and the buried surfaces of SET-I and SET-N and re-establishes a functional SET domain. As a result of these interactions, the SET-I α-helix rotates in a counterclockwise manner around two conserved “hinge” Gly residues (Gly643 and Gly681) and returns to its active orientation (green structure in Figure 2D).^63^ The substrate binding site is further released by an outward torsion of the post-SET region around its loop structure (residues 726–729) that simultaneously clears the lower part of the histone-recognizing channel and shapes the lid of the cofactor binding pocket (green structure in Figure 2E).^23,63^ These conformational changes together with the recognition of SAL-like structures in other SET-containing methyltransferases affirm the SAL domain as a key effector in mediating the SET transition toward an active catalytic state.^63^

**Figure 3:**
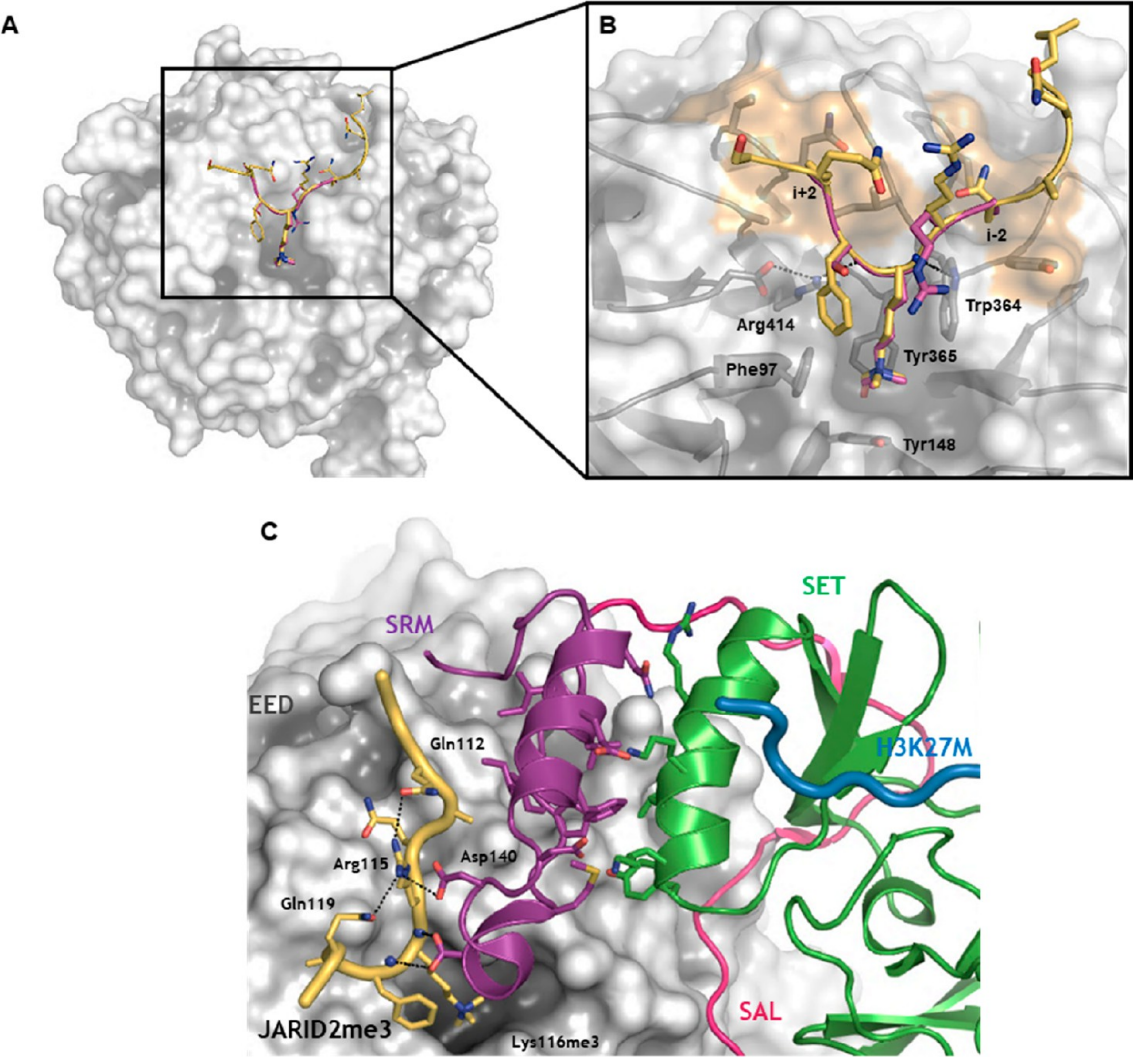
Allosteric binding site and activation of the PRC2 core complex. (A) JARID2me3 and H3K27me3 peptides binding to the EED central cavity. EED is represented in gray surface, and JARID2me3 (PDB 5HYN) and H3K27me3 (PDB 3IIW) are in yellow and magenta cartoons, respectively. (B) Enlarged view of H3K27me3 peptide superimposed to JARID2me3 cocrystallized with EED (PDB 5HYN). Peptide side chains are presented as sticks; EED is in semitransparent surface and cartoon. Amino acids delimiting the trimethyllysine aromatic cage are represented as sticks and labeled. Molecular hollows hosting the *i* + 2 and *i* – 2 amino acids from trimethylated lysine are highlighted in orange surface. Hydrogen bonds are in dashed black lines. (C) Allosteric stimulation of the SET catalytic function. EED is in gray surface, JARID2me3 peptide (yellow), SAL domain (magenta), SRM (violet), SET (green), and H3K27M peptide (blue) are in cartoon representation with key residues labeled and in sticks. Hydrogen bonds are shown in dashed black lines.

Akin to the bottom side, the top surface of EED supplies additional sites of interaction with EZH2, and through the recognition of a quaternary ammonium moiety in its central cavity, it modulates the SET catalytic function in a context-specific manner. In this respect, Margueron et al. first reported a cocrystal structure of isolated EED with a H3K27me3 peptide embedded within the top gorge of the β-propeller, thus providing structural insights on its epigenetic “reading” function.^26^ This recognition is not limited to the H3K27me3 mark, and other trimethyllysine-containing ligands were discovered to bind to EED (Figure 3A).^7,26,27^ Nonetheless, not all of these histone-derived binders prompted an allosteric stimulation *in vitro*, and only H3K27me3 and JARID2me3 proved to increase, up to 3-fold, the nucleosomal methyltransferase activity of PRC2.^7,27^ Despite these differences, the EED recognition of trimethylated ligands relies on common binding features, revealed here by overlaying the poses of H3K27me3 and JARID2me3 within the shallow pocket of the β-propeller structure (Figure 3B).^7,26^ The ligands dock by their trimethylammonium moieties, which interact with a triad of aromatic amino acids (Phe97, Tyr148, and Tyr365) whose almost perpendicular arrangement defines the so-called “aromatic cage”. Besides these π–cation juxtapositions, the trimethyllysine side chains assume an extended conformation and position van der Waals interactions with the nearby Trp364 indole ring, which also interacts by polar contacts with the peptide backbones. Finally, the H3K27me3 and JARID2me3 are further stabilized on the EED surface by hydrogen bonds occurring between the rim of the cavity (Arg414) and the main-chain carbonyl groups flanking the Lys27me3 and Lys116me3. Since the aromatic cage organization is a structural trait common to other epigenetic “readers” (i.e., chromodomain proteins), the EED selectivity toward H3K27me3 and JARID2me3 can be explained considering two additional shallow hydrophobic ditches surrounding the central gorge of EED (highlighted in orange surface) where they orient van der Waals interactions through small aliphatic amino acids at *i* – 2 and *i* + 2 positions relative to the trimethylated lysine.^12,64−66^ Indeed, both of them contain an Ala residue at *i* + 2 position, whereas at the *i* – 2, the H3K27me3 peptide presents an additional Ala and JARID2 contains a Gln, which can recline to orient its aliphatic side chain to the local hydrophobic surface of EED (Figure 3B).^7^

The mechanism of the allosteric activation of EZH2 catalysis takes advantage of the role of EED as mentioned earlier and has been elucidated at an atomic level in *Chaetomium thermophilum* and human trimeric PRC2, in complex with H3K27me3 and JARID2-K116me3 stimulating peptides, respectively.^59,60^ Both of them merged to a model centered on the role of the EZH2-SRM domain in communicating the allosteric inputs from the EED top surface to the SET catalytic region. In this dynamic, the EZH2 arrangement around EED brings the disordered SRM to a location near the β-propeller cavity, where it senses the presence of stimulating signals. In a PRC2 ternary complex at the basal state, the SRM domain is disordered and unresolved in the electron density map.^60^ However, when H3K27me3 or JARID2-K116me3 bind to EED, a set of side-chain contacts between these allosteric modulators (we show here JARID2-K116me3) and the SRM itself determine its conformational stabilization in an α-helical structure. This interaction is dominated by Arg115_(JARID2)_ (or Arg26 in H3K27me3 peptide), which is an invariant feature of repressive trimethyllysine histone marks and establishes polar contacts both with the SRM helix (Asp140) and intramolecularly with Gln112 and Gln119 (Figure 3C). This ordered disposition causes the juxtaposing surfaces of the SRM and SET-I α-helices to tightly pack to each other and to converge to an overall sandwich-like arrangement, which is supposed to be responsible for the increased catalytic activity of the SET domain.

## 3 Inhibitors of EZH2–EED Protein–Protein Interaction

Considering the multiproteic nature of the PRC2 complex and the marked dependence of EZH2 functions on the other core subunits, in recent years, a new targeting approach ascended to prominence. The possibility to cripple the function of the PRC2 complex by interfering with its multimeric integrity fueled interest in developing protein–protein interaction (PPI) inhibitors as indirect anti-EZH2 agents. In this respect, a seminal study reported in 2013 the development of an all-hydrocarbon stapled peptide recapitulating the α-helical EED-binding domain (EBD) of EZH2 and disclosed the feasibility of hampering the interaction between EED and EZH2 to disable the PRC2 biological functions.^67^

The stabilized α-helix of EZH2 (SAH-EZH2) selectively inhibits H3K27 trimethylation by dose-responsively disrupting the EZH2–EED complex, evaluated through fluorescence polarization (FP) assay, and reducing EZH2 protein levels. Notably, the reduction of EZH2 protein level was not observed upon treatment with GSK126. The impairment of the EZH2–EED interaction led to proliferation arrest and myeloid differentiation in PRC2-dependent MLL-AF9 leukemia cells and remarkably affected the viability of cancer cells bearing EZH2 mutant variants. Given the two distinct mechanisms of action between a catalytic EZH2 inhibitor (GSK126) and the SAH-EZH2 peptide, the authors investigated whether the coadministration of the two compounds would enhance the antiproliferative effects. Indeed, upon the cotreatment of MLL-AF9 and KARPAS422 cells with SAH-EZH2 and GSK126, an apparent synergistic impairment of cell viability was observed.^67^ Although peptide-based chemotypes are often characterized by a poor pharmacokinetic (PK) profile, Orkin and co-workers demonstrated the therapeutic potential of targeting the binding interfaces between the PRC2 partners as a novel strategy to modulate their biological functions and paved the way for the development of small molecule PPI inhibitors.^67^

In a following study published in 2014, the crystal structure of EED in complex with an EZH2-EBD-resembling peptide (Figure 5A, PDB 2QXV) was used for docking-based virtual screening of an in-house database containing approximately 1000 known drugs by Luo’s research group.^68^

**Figure 4:**
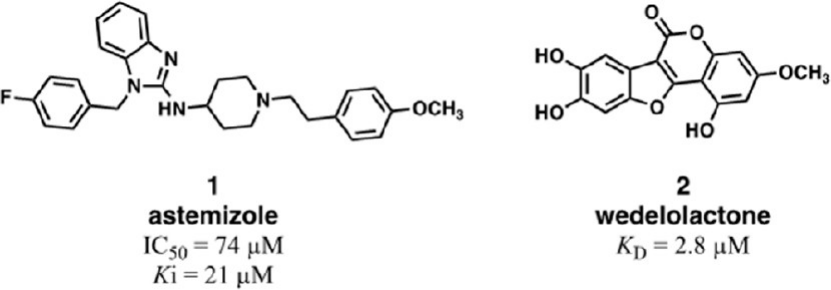
Structures of astemizole (**1**) and wedelolactone (**2**).

Among them, astemizole (**1**, Figure 4), a second-generation FDA-approved (but later withdrawn from the market) antiallergy drug with histamine H1 receptor antagonist activity,^69^ showed the highest EZH2–EED interaction inhibitory activity, displacing the EZH2 peptide with a *K*_i_ of 23.01 μM in a competitive fluorescence polarization assay. To validate whether **1** directly bound to EED and competed with EZH2, additional methods, including fluorescence-based thermal shift and saturation transfer difference (STD) nuclear magnetic resonance (NMR) spectra, were used, and the results corroborated the first experiments. Considering the importance of the EZH2–EED interaction for the integrity of the PRC2 complex, **1** was also evaluated for assessing its capability to reduce PRC2 protein levels upon dissociation of the EZH2–EED complex. As expected, **1** treatment dose-dependently decreased the protein levels of EZH2, EED, and SUZ12 in both the DB (EZH2 mutant) and Toledo (wild-type EZH2) cell lines, which was in agreement with the reported effects of mutations able to disrupt these protein–protein interactions.^24,70^ Furthermore, low micromolar concentrations of **1** markedly impaired the proliferation of GCB-DLBCL cells carrying mutant or wild-type forms of EZH2. Of note, a synergistic effect was observed in both DB and Toledo cells upon the coadministration of **1** and the catalytic EZH2 inhibitor EPZ005687, corroborating the results observed for the stapled EZH2 peptide in the study above-mentioned.^67^

In 2015, a different work described the discovery of the natural compound wedelolactone (**2**, Figure 4) being able to bind to EED with a micromolar affinity (*K*_D_ = 2.82 μM), to block the EZH2–EED interaction in vitro, to induce the degradation of both EZH2 and EED proteins at 50 μM in HepG2, THP1, and K562 cells, and also to modulate the expression of detected PRC2 downstream targets and cancer-related genes.^71^

**Figure 5:**
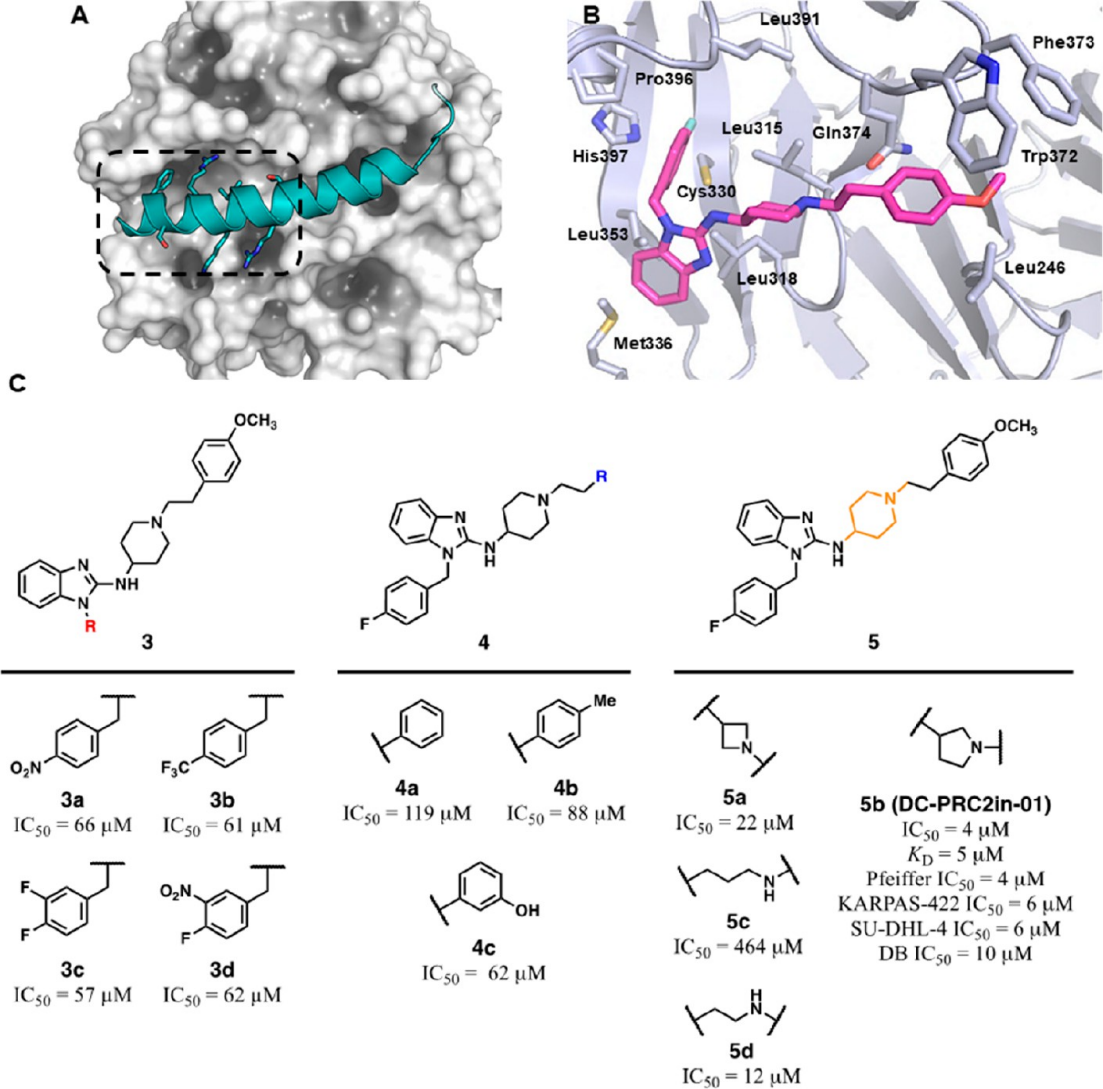
X-ray cocrystal structures of EZH2 EBD domain and astemizole in complex with EED and structural optimization of astemizole-derived compounds (**3**–**5**). (A) X-ray crystal structure (PDB 2QXV) of EBD peptide in cartoon (teal) with EED in surface (white). The dashed square represents the EBD-binding site portion where astemizole is accommodated. (B) Binding mode of astemizole, in stick (magenta) along the bottom surface of EED, in cartoon (blue-white). The amino acids important for the binding are represented as sticks and labeled. (C) Structural optimization of astemizole-like compounds (**3**–**5**) and leading to DC-PRC2in-01 (**5b**).

Very recently, Luo’s group published the cocrystal structure of EED in complex with **1** at 2.15 Å (residues 40–441) (PDB 7KXT).^72^ The structure (Figure 5B) elucidates the detailed binding mode of **1** to EED and provides insights for a structure-guided drug design that led to a novel EZH2–EED interaction inhibitor, DC-PRC2in-01 (**5b**). The compound **5b**, bearing a pyrrolidine moiety instead of the piperidine as in **1**, displayed a *K*_D_ value versus EED protein of 5 μM, measured by surface plasmon resonance (SPR), and an IC_50_ of 4 μM, determined by fluorescence polarization competitive binding assay. As shown in Figure 5B, EED folds into a typical seven-bladed β-propeller structure with **1** binding to the bottom of the WD40-repeat domain, where EED recognizes the EBD domain of EZH2 (residues 32–70) (Figure 2B,C, see above),^61^ and differently from the EED binders that dock in the H3K27me3 binding pocket at the top of WD40-repeat domain of EED.

The binding site accommodating **1** extends along the EED cleft that harbors the N-terminal portion of the EZH2-EBD domain (dashed square in Figure 5A) and involves a series of mostly hydrophobic residues. Compound **1** binds to this site with the fluorobenzene group inserting deeply into a hydrophobic pocket formed by a cluster of 6 residues (Leu315, Cys330, Leu391, Val393, Pro396, and His397) (Figure 5B). Compared with the structure of the EZH2-EBD peptide bound to EED, this fluorobenzene group occupies the space of the hot-spot residue Phe42 of EZH2 (Figure 5A).^61^ Moreover, the benzimidazole moiety establishes interactions with hydrophobic residues Leu318, Met336, and Leu353, whereas the 4-methoxyphenethyl moiety engages in hydrophobic interactions with Leu246, Phe372, Trp373, and Gln374.^72^

The compound **1** scaffold was optimized according to two lines of intervention: (1) the *N*-benzyl and the terminal phenethyl moiety were modified with functional groups aiming to reinforce the hydrophobic interactions or to establish novel polar contacts within the connectivity of the original structure (**3** and **4**); (2) the distance between the nitrogen atoms of the 4-aminopiperidine linker was optimized through cyclic homology and ring-opening study of the piperidine moiety (**5**) (Figure 5C).

Among the *N*-benzyl modifications, only the *para*- and the *meta*,*para*-disubstitution with electron-withdrawing groups (EWGs) proved to be tolerated (**3a**–**d**), yielding a weak increase of potency (IC_50_ = 57–66 μM) with respect to **1** (IC_50_ = 74 μM), while on the phenethyl terminal only the *meta*-hydroxyl modification (**4c**) provided a similar improvement (IC_50_ = 62 μM). Manipulation of the 4-aminopiperidine moiety provided a significant improvement in the potency and highlighted the importance of maintaining a proper length and geometry in this portion (**5a**–**d**). In this regard, the piperidine ring replacement with pyrrolidine afforded compound **5b** (DC-PRC2in-01), showing a 17-fold increase of potency (IC_50_ = 4 μM). This result was rationalized by molecular docking studies that hypothesized the formation of H-bonds between the 3-aminopyrrolidine nitrogen atoms and the surface of EED. Furthermore, while the cyclic homologation to azetidine (**5a**) still provided a more potent compound (IC_50_ = 22 μM) than **1**, the piperidine ring-opening led to inactive compound (**5c**) whereas the pyrrolidine ring-opening (**5d**), bearing an ethylene spacer, was tolerated.

At a protein level, **5b** was able to destabilize the PRC2 complex, thus leading to the dose-dependent depletion of mainly EED along with EZH2 and SUZ12 proteins up to 10 μM. Notably, **5b** dose-dependently and specifically reduced the levels of H3K27me3 with respect to other H3 methyl marks, thus demonstrating the efficacy of the EZH2–EED interaction inhibitors in evoking epigenetic effect.^72^ Compound **5b** dose-dependently impaired cell viability of Pfeiffer, KARPAS422, SU-DHL-4, and DB cell lines, harboring EZH2 heterozygous mutations, with IC_50_ values below 10 μM, by blocking the cell cycle in G0/G1 phase.

This study paves the way to (a) a new in-depth astemizole SAR investigation for increasing its potency and (b) the design of novel molecular models for disrupting the EZH2–EED interaction.

## 4 First Steps toward EED Binders

**Figure 6:**
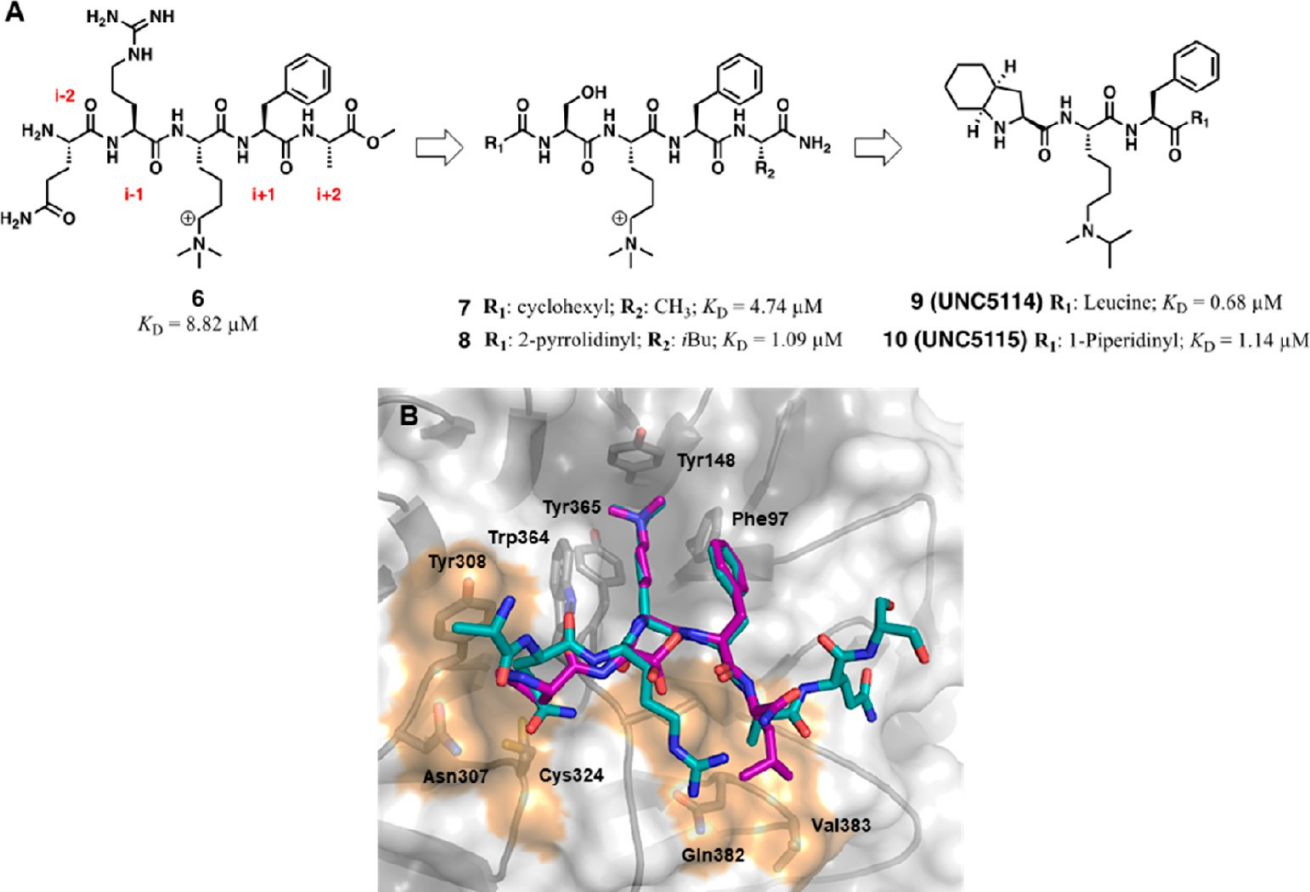
Chemical structure of JARID2-K116me3-inspired peptidomimetics and binding pose comparison between JARID-K116me3 and compound **8**. (A) Evolution of SAR studies on 5-mer JARID2-K116me3 structure leading to **9** (UNC5114) and **10** (UNC5115). (B) Superimposition between structures of 13-mer JARID2-K116me3 (teal, PDB 4X3E) and **8** (violet, PDB 5TTW) in stick representation and complexed with EED (in gray surface and cartoon representations). EED interacting amino acids are labeled and represented as sticks. Polar contacts are represented as dashed black lines. Surfaces harboring chemical entities in *i* – 2 and *i* + 2 positions are highlighted in light orange.

The evidence that trimethylated JARID2 protein binds to EED and allosterically stimulates the PRC2 methyltransferase activity prompted the initial attempts to address the EED top cavity for targeting the PRC2 functions.^7^ In this regard, Barnash et al. exploited a previously developed combinatorial peptide screening platform to readily generate a set of peptidomimetic EED binders inspired by JARID2-K116me3, eventually steering toward a 4-mer micromolar PRC2-allosteric inhibitor, UNC5114 (Figure 6A).^73,74^

The 13-mer JARID2-K116me3 peptide represented an advanced starting point in the optimization process since it exhibits a 10-fold higher affinity for EED compared with H3K27me3 *in vitro* (*K*_D_ = 3 versus 40 μM, respectively) and it primarily interacts through five amino acids (QRKme3FA, **6**) centered on the K116me3 with Gln114 and Ala118 providing auxiliary interactions (Figure 6A,B).^7^ With the aim to move toward a more druglike scaffold and revert adverse physicochemical properties, such as an overall +3 charge and the presence of disadvantageous polar functions, peptide **6** underwent two different rounds of combinatorial modifications. First, the *i* – 2 position was varied with apolar substituents able at the same time to mimic the Gln114 and to cap the cationic N-terminus. These chemical manipulations led to the identification of compound **7** that in isothermal titration calorimetry (ITC) measurements showed a 2-fold improvement (*K*_D_ = 4.74 μM) with respect to **6**. Second, a more robust round of optimization encompassed a larger portfolio of replacements and included the *i* + 2 position as variable element. A combination of the beneficial modifications into a common structure resulted in **8** exhibiting a further enhancement in potency (*K*_D_ = 1.09 μM) and decreased +2 overall charge. Compound **8** was cocrystallized with EED and preserved the binding pose adopted by the 13-mer JARID2-K116me3 (Figure 6B), with the terminal Leu and Pro residues harbored in the same hydrophobic hollows hosting the Ala118 and the Gln116 from JARID2 peptide, respectively. Consistent with the negligible contribution to the β-propeller binding, the *i* – 1 position can hold a certain degree of variability, with the Ser side chain being unresolved and pointing toward the solvent.

The understanding of the structural determinants responsible for the **8**–EED interaction assisted the last stage of the optimization toward a peptidomimetic structure. Indeed, the ensuing modifications revolved around the shortening of the amino acidic sequence and the replacement of the trimethyllysine moiety with a more cell-permeable function. In this respect, the substitution of the K116me3 cationic function with an *N*-methyl-*N*-isopropyl amine, together with the Pro-Ser dimer replacement with the bicyclic (2*S*,3a*S*,7a*S*)-octahydro-1*H*-indole afforded the tetrameric UNC5114 (**9**), featuring a sub-micromolar affinity (*K*_D_ = 0.68 μM) and fewer amide bonds. Insertion of a piperidinyl nucleus in place of the C-terminal Leu brought about a cognate compound of **9**, namely, UNC5115 (**10**) exhibiting a lower affinity (*K*_D_ = 1.14 μM).

To shed light on the effect on PRC2 methyltransferase activity, the UNC5114 and UNC5115 were functionally evaluated in a scintillation proximity assay where they proved to decrease the catalytic activity up to 20% of the saturating concentration of ligand. Additional biochemical evaluations validated the allosteric mechanism of action for **9** and **10**, and their strict dependence on the aromatic cage recognition for binding was substantiated by the loss of PRC2 inhibition in F97A, W364A, and Y365A EED mutants.

## 5 Discovery of Hit Compounds as EED Binders

**Figure 7:**
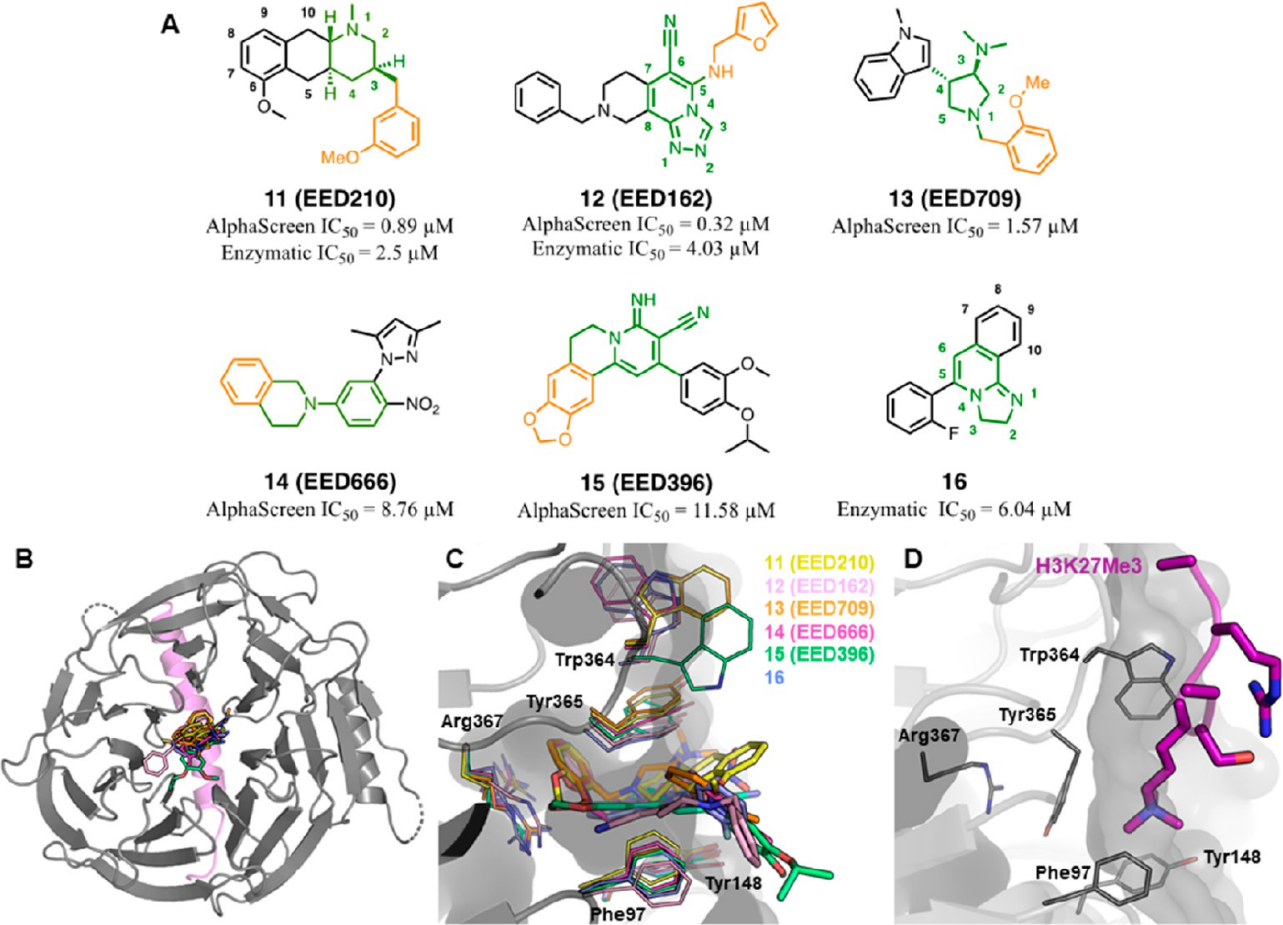
Chemical structures of identified EED binders (**11**–**16**). (A) Chemical structures of compounds **11**–**16** showing the moieties exploring the “deep pocket” highlighted in orange and the scaffolds interacting with the aromatic cage highlighted in green; (B) Overall structure of EED–EBD–(**11**–**16**) ternary complex; EED is represented in semitransparent surface and cartoon (gray) (PDB 5H19), EBD peptide is represented in cartoon (violet), and hit compounds are represented in differently colored sticks. (C) Binding modes of **11**–**16** within the H3K27me3 pocket of EED (gray). Structures are shown as stick representations within X-ray cocrystal structure of **11** with EED (PDB 5H17). The residues delimiting the sides and the bottom of the binding gorge are labeled and represented as thin sticks. (D) Binding mode of H3K27me3 peptide (violet cartoon) within the upper cavity of EED (in gray cartoon and surface) (PDB 3IIW). The H3K27me3 side chains are represented as sticks. PDB codes for the other hit compounds: **12** (PDB 5H19), **13** (PDB 5H15), **14** (PDB 5H14), **15** (PDB 5H13), and **16** (PDB 5H25).

First reports on hit identification of small molecular entities as EED binders were released by Novartis researchers in 2017. The development of a homogeneous time resolved fluorescence (HTRF) assay for a five-component PRC2 complex enabled the high-throughput screening (HTS) of approximately 1.4 million compounds.^75^ The assay was based on the detection of H3K27me0 to H3K27me2 conversion of a substrate peptide (histone H3, residues 21–44) and used a europium cryptate labeled antibody as a FRET donor. Given the inability of this preliminary test to discriminate between SAM competitive and noncompetitive PRC2 inhibitors, different biochemical investigations were carried out to define the mechanism of action of the identified hits. Compounds with IC_50_ < 50 μM were first evaluated in the same HTRF assay using different SAM concentrations to ascertain the activity cofactor independence. Then, the allosteric nature of the PRC2 inhibition was confirmed, measuring the binding disruption between a resin-bound EED protein (residues 1–441) and a H3K27me3 peptide (residues 19–33) in an AlphaScreen format. By means of this biochemical crossroads, different low-micromolar range H3K27me3-competitive PRC2 inhibitors were identified, belonging to different scaffolds, and a representative set of these (**11**–**16**) was disclosed (Figure 7A).^76^ Alternatively, in 2019, researchers from AstraZeneca developed a fluorescence polarization-based assay to identify EED binder hits with a single run of experiments readily. Their assay employed a previously reported EED binder as a displacement probe with the advantage of leading to hits with the same mechanism of action and less unspecific binding events.^77,78^

Compounds **11**–**16** were crystallized in complex with EED and an α-helical strand resembling the EZH2-EBD domain (residue 40–68). These cocrystal structures revealed that the compounds dock in the H3K27me3 binding pocket of EED (Figure 7B), albeit relying on an “induced fit” in the aromatic cage compared to the H3K27me3-bound configuration (Figure 7C,D). Some of the key residues governing the width of the cavity (Tyr364 and Trp365) were engaged in relevant side chain rotations to accommodate the diverse structures of **11**–**16**, while Phe97 and Tyr148 were unperturbed. Similar molecular movements were also observed for Arg367, which in H3K27me3-bound EED is shielded by Tyr365 but with EED binder interaction lies at the bottom of the cavity and swings between two different conformations. The combination of movements of Tyr365 and Arg367 determines the loss of their reciprocal π–cation stacking interaction, which is a feature of the apo-structure of EED. Arg367 does not shape the wall of the aromatic cage; however, it fulfils the role of “gatekeeper” residue for the access to the interior of the EED β-propeller hole, thus defining an additional site of interaction, referred to as “deep pocket” or “induced pocket”. The observed conformational changes between the inhibitor-bound and the apo-structures of EED evidenced a certain adaptability of the β-propeller cavity in modeling its width and depth toward different scaffolds and furnished an explanation for the chemical diversity of the binders identified by Li et al.^75^

Compounds **11**–**16**, although displaying different structural cores, shared several points of interaction and the overall orientation within the binding site. With the sole exception of **16**, all the compounds are endowed with electron-rich aromatic systems (highlighted in orange in Figure 7A) to interact with the Arg367 guanidinium group by cation−π stacking in a face-to-face (**11**–**14**) or edge-to-face orientation (**15**). The central scaffolds of **11**–**16** (highlighted in green in Figure 7A), despite not being rationalizable in a common pharmacophore, locate within the space housing the trimethylated lysine of H3K27 and establish π–π stacking (**12** and **14**–**16**) or cation−π and van der Waals interactions (**11** and **13**) with the amino acids outlining the sides of the aromatic cage (Phe97, Tyr365, and Tyr148). The adaptability of the allosteric binding site also reflects the possibility of granting distinct interactions between different functional groups of **11**–**15** and ancillary amino acids projecting on the inner surface of the H3K27me3 binding cavity (see ref (91) for further details). The optimization of these contacts was exploited to improve the binding affinity and to develop more efficient EED binders starting from the hits **11**–**13**.

### 5.1 Structural Optimization of EED210

**Figure 8:**
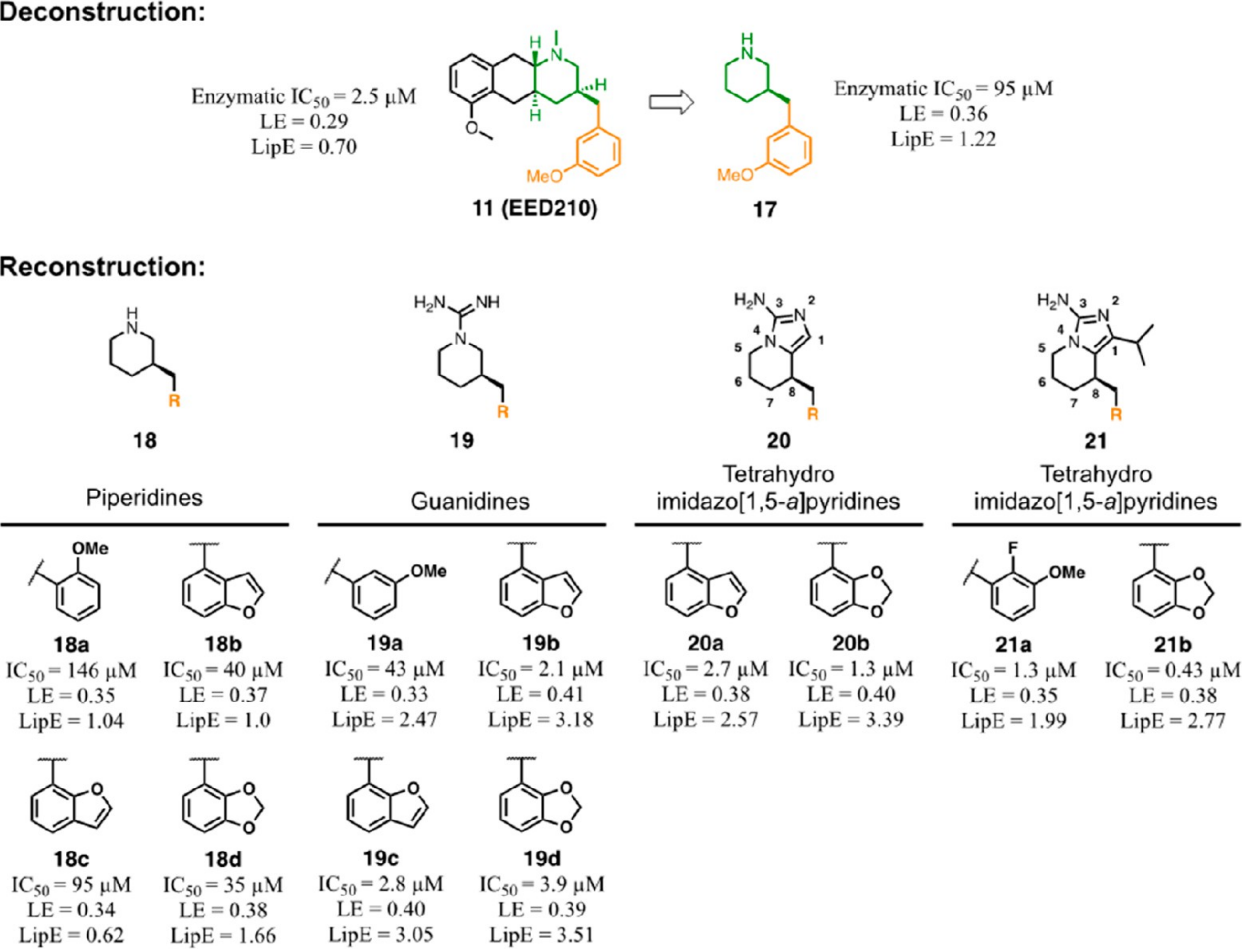
Deconstruction and reconstruction of **11** (EED210) and its derivatives (**17**–**21**). LE and LipE indexes were calculated according to the following formula: LE = (1.36 × pIC_50_)/HAC and LipE = pIC_50_ – cLogP. HAC = heavy atom count.

In the wake of the hit discovery campaign reported by Li et al.,^75^ the H3K27me3-competitive PRC2 inhibitor EED210 (**11**, Figure 7A) was selected for further optimization.^79^ EED210 exhibited a modest micromolar inhibition of the PRC2 complex in the enzymatic assay (IC_50_ = 2.5 μM), and in the cocrystal structure with EED, it docked in the H3K27me3-binding cavity with an interaction mode similar to that observed for the other identified hits (Figure 7C).^75^ With the aim to optimize the binding efficiency and to converge to a synthetically more accessible scaffold, EED210 underwent structural manipulations according to a deconstruction–reconstruction approach (Figure 8). Preservation of the pharmacophoric elements along with simplification of the tricyclic core of EED210 led to the piperidine-based compound **17**. Despite its essential structure, **17** retained the ability to inhibit PRC2 (IC_50_ = 95 μM) and afforded an increase in ligand efficiency (LE) and lipophilic efficiency (LipE) with respect to the parent compound **11**, thus indicating an improved balance between fragment affinity and lipophilicity.^80,81^

**Figure 9:**
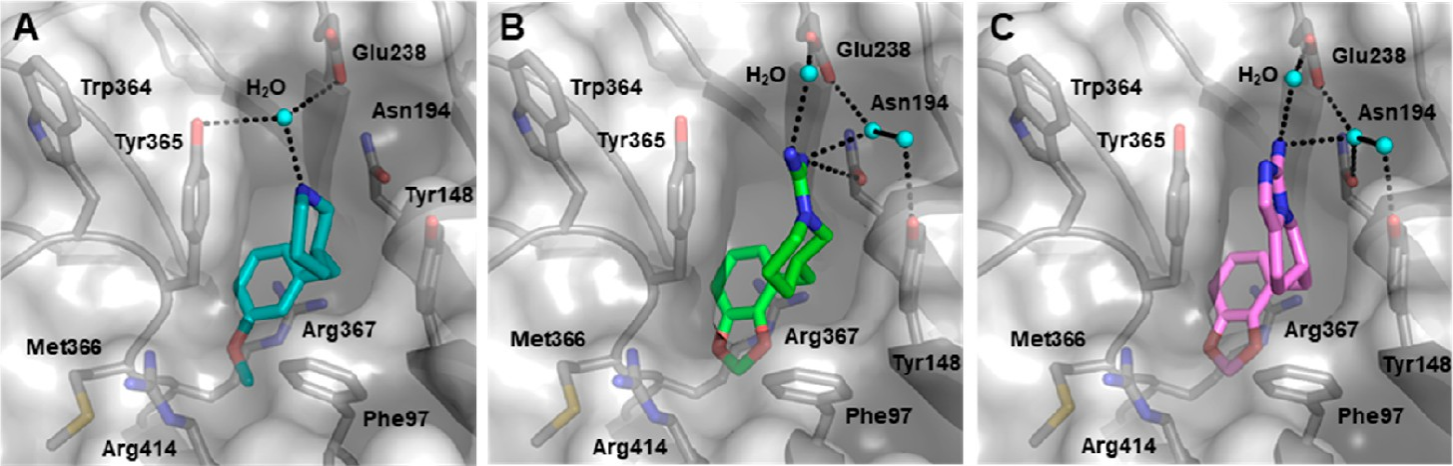
X-ray cocrystal structures of **17**, **19d**, and **20b** within EED structure. (A) Binding mode of **17** (teal) within the H3K27me3 pocket of EED (gray); interacting residues are labeled and represented as sticks (PDB 5U5K). (B) Binding mode of **19d** (green) within the H3K27me3 pocket of EED (PDB 5U5T). (C) Binding mode of **20b** (violet) within H3K27me3 pocket of EED (PDB 5U62). In all three cases, water molecules are labeled and reported as cyan balls; inter- and intramolecular H-bonds are represented as dashed black lines.

These results found confirmation in structural analysis (Figure 9A) where **17** was shown to retain the ability to embed within the remodelled H3K27me3 binding site and its 3-methoxyphenyl portion leveraged deep-pocket interactions with the Arg367. Interestingly, the protonated piperidine ring perfectly docked within the aromatic cage where it mimicked the presence of the H3K27me3 quaternary amine and contacted the Tyr365 and Glu238 by water-mediated H-bonds. From this minimized fragment, the authors started a stepwise reconstruction process to elicit improved chemical–physical properties, which involved the methoxyphenyl moiety and the core interacting with the aromatic cage (Figure 8). In this concern, given the relevance of π–cation interactions with the Arg367 guanidinium group for efficient binding, the deep pocket was probed with electron-rich aromatic systems characterized by the methoxy group shifted in different positions or where the oxygen atom was included in a bicyclic core (**18a**–**d**). Among these, the methoxy group in *meta* (**17**) or in *ortho* (**18a**) position represented the sole tolerated options, whereas the replacements with benzofuran regioisomers (**18b**,**c**) or benzodioxole ring (**18d**) afforded up to a 2-fold increase or retention of enzymatic potency (IC_50_ = 35–95 μM) together with improved LE and LipE.

Subsequently, attention was paid to the possibility of replacing the point charge-mediated π–cation stacking with interactions arising from a charge delocalized on π-aliphatic or π-aromatic systems. Maintaining the previous beneficial deep-pocket moieties, the piperidine secondary amine was converted into a guanidine group or fused within a tetrahydroimidazo[1,5-*a*]pyridine (Figure 8). Combining the π-aliphatic system with selected deep-pocket moieties resulted in a general increase of the PRC2 inhibition, especially for the benzofuran- or benzodioxole-bearing compounds (**19b**–**d**), which were characterized by single-digit micromolar range potency (IC_50_ = 2.1–3.9 μM). Importantly, these compounds showed a significant improvement in terms of lipophilic efficiency (LipE = 3.05–3.51) that reflects a better exploitation of direct contacts rather than unspecific lipophilic interactions to increase ligand binding. The cocrystal structure of **19d** with EED (Figure 9B) explained the observed improvements and revealed that the guanidinium group, while juxtaposed with Tyr148 and Tyr365, facilitates H-bonds with Tyr148, Asn194, and Glu238, either directly or via a water molecule. These polar contacts teamed up with the deep-pocket interactions featured by the parent fragment **17** and were responsible for the remarkable increase of the binding potency. In ensuing assessments, **19a**–**d** proved to inhibit the H3K27 methylation in kidney cancer (G-401) cells. However, due to permeability issues, they did not proceed in further development. Cellular permeation is negatively affected by strong positive charge; therefore, it was speculated that selecting moieties with appropriate p*K*_a_ values might provide a guideline to properly mimic the H3K27me3 cationic nature without dampening the permeation capability. Looking for a protonable source with π aromatic delocalization potential, the authors reported a following series of compounds bearing a 3-amino- (**20a**,**b**) or 1-isopropyl-3-amino-tetrahydroimidazo[1,5-*a*]pyridine (**21a**,**b**) (Figure 8). This choice allowed a reduction of the number of H-bond donors and mitigated the high p*K*_a_ values disclosed by the previous series of compounds (guanidine p*K*_a_ > 11 versus aminoimidazole p*K*_a_ = 9.2). When tested in an enzymatic setup, these compounds preserved their inhibitory activities in single-digit (**20a**,**b** and **21a**) to sub-micromolar range (**21b**) and importantly demonstrated a marked enhancement of cell permeation in a cellular Caco-2 model. Inhibitory effects on H3K27 methylation status in G-401 cells were similarly maintained, and notably, the sub-micromolar inhibitor **21b** showed a dose-dependent antiproliferative effect in Pfeiffer cells (IC_50_ = 3.4 μM), thus proving that allosteric inhibition of EED might impair cellular growth in lymphoma cells. EED–**20b** binary complex (Figure 9C) provided structural insights to the presumable binding pose of **21b** and, in general, for this series of compounds. Compound **20b** engages the same deep-pocket interactions of its parent compound **19d** employing the benzodioxole ring and fills the aromatic cage triad through its 5,6,7,8-tetrahydroimidazo[1,5-*a*]pyridine core that establishes face-to-face π–cation stackings with Tyr148 and Tyr365. Contrarily, the amino group of **20b** could not protrude to directly contact Asn194 and Glu238 polar groups and interacted with these moieties via a water molecule network. Finally, in the EED–**21a** cocrystal structure (reported by the authors but not included in the present review), the 4-isopropyl pendant bulged outside the EED cavity toward the solvent and was accounted as a plausible position for further future modifications.^79^

### 5.2 Structural Optimization of EED162

**Figure 10:**
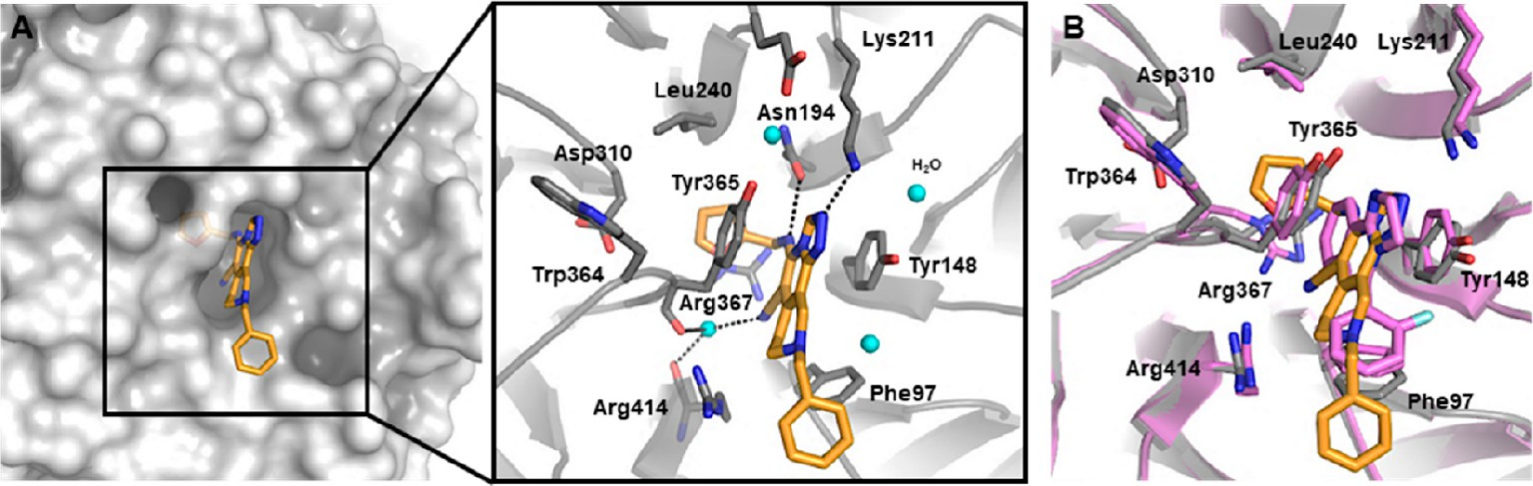
Binding interactions and X-ray crystal structure of EED–EBD–**12** (EED162) and EED–EBD–**16** ternary complexes. (A) Binding mode of **12** is presented in orange sticks within the H3K27me3-binding pocket of EED in white surface (on the left) and polar contacts within EED aromatic cage (on the right) (PDB 5H19). Interacting amino acids are labeled and shown in gray stick representation, whereas water molecules are depicted as cyan balls, and inter- and intramolecular H-bonds are represented as dashed black lines. (B) Superimposition of binding modes of **12** and **16** within the cavity of EED. Compound **16** is represented in pink; the corresponding EED cocrystal structure is in a pink cartoon (PDB 5H25), and the amino acids outlining the rim of the pocket are labeled and represented as sticks.

**Figure 11:**
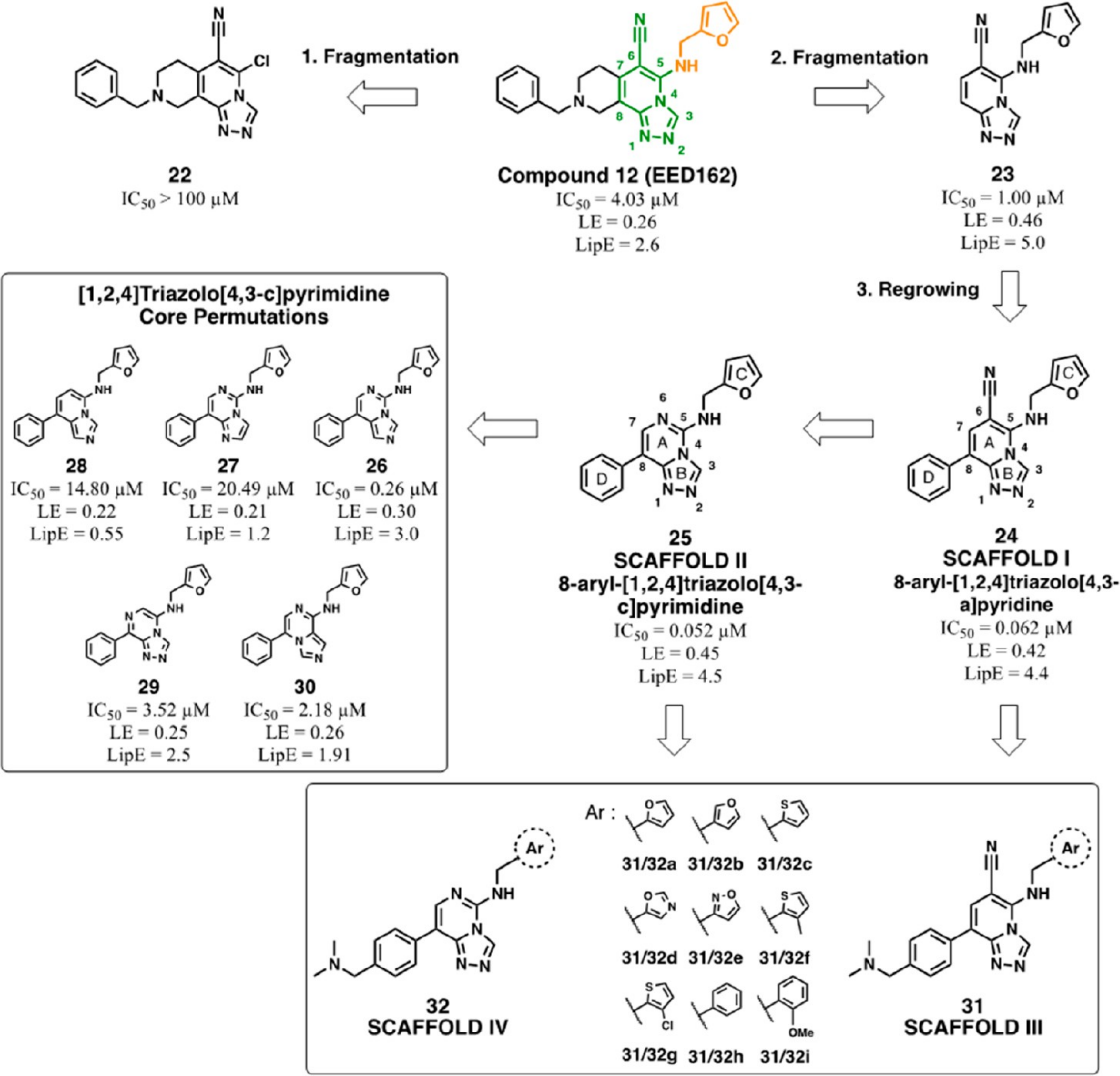
Overview of structural optimizations of compound **12** (EED162). Evolution of SAR investigations on compound **12** leading to scaffold I (8-aryl-[1,2,4]triazolo[4,3-*a*]pyridine (**24**)), and scaffold II (8-aryl-[1,2,4]triazolo[4,3-*c*]pyrimidine (**25**)), followed by further optimizations and definition of scaffold III (**31**) and IV (**32**). Ligand efficiency (LE) and lipophilic efficiency (LipE) indexes were calculated according to the following formula: LE = (1.36 × pIC_50_)/HAC and LipE = pIC_50_ – cLogP. HAC = heavy atom count.

In a further investigation, Huang et al. focused on compound **12** (EED162) and started a structure-based hit optimization campaign on its core structure.^82^ The high-resolution cocrystal structure (PDB 5H19) of EED162 revealed that the binding into the H3K27me3 pocket relies on the establishment of a set of π–π interactions and hydrogen bonds with the EED counterpart (Figure 10A). An aromatic face-to-face π–π juxtaposition between the electron-deficient [1,2,4]triazolo[4,3-*a*]pyridine and the electron-rich Tyr148 and Tyr365 of the aromatic cage held in place the central scaffold of **12** in the middle of the β-propeller pocket. The so-obtained binding geometry enables the furan-2-yl in C5 of the core to dock into the “deep pocket” where the furan ring positions cation−π and edge-to-face π–π interactions with the guanidinium group of Arg367 and Tyr365, respectively. This molecular portion penetrates into a predominantly hydrophobic region of EED, outlined by Leu240, Asp310, and Arg367, where the 5-aminomethylene linker faces the entrance of the “induced pocket” and undergoes polar contacts with the Asn194 side chain. Other structural elements additionally contribute to the interaction: the endocyclic N2 nitrogen affords a hydrogen bond with Lys211 on the rim of the H3K27me3 pocket, whereas the cyano group in C6 interacts with the backbone of Tyr365 and Arg414 via water-mediated H-bonds. Remarkably, the C7/C8-fused *N*-benzylpiperidine is not essential for the binding and confers a detrimental lipophilicity to a solvent-exposed molecular portion, thus representing a suitable position for modification in a deconstruction–reconstruction approach.^82^ Initial fragmentation of the EED162 structure led to the compounds **22** and **23** (Figure 11), lacking the furan-2-yl-methylenamine in C5 and *N*-benzylpiperidine in C7–C8, respectively, and demonstrated the importance of leveraging “deep pocket” interactions. Indeed, while **23** (IC_50_ = 1 μM) retained the same activity range of **12** (IC_50_ = 4.03 μM) in biochemical assay, compound **22**, which was unable to penetrate deeply into the inner H3K27me3 pocket, was inactive (IC_50_ > 100 μM).

Structural analysis of the HTS hit **16** (Figure 10B) suggested that an edge-to-face π–π stacking between its C5 phenyl ring and Phe97 might provide a beneficial interaction for the affinity. This compound, even though devoid of the pivotal structural features seen in **12**, such as an electron-rich aryl group in C5 position and an H-bond acceptor in N2, elicited a single-digit micromolar inhibition of the EED–H3K27me3 interaction (IC_50_ = 6.04 μM) comparable to that of **12** (Figure 7A). Superimposition of the crystal structures of **12** and **16** revealed that an aryl substitution in the C8 position of **12** might correspond to that shown in C5 by compound **16** (Figure 10B). As a result, the combination of structural features from compounds **16** and **23** led to the structure **24**, characterized by a 5,8-disubstituted bicyclic aromatic module (scaffold I, Figure 11) and displaying a 60-fold improvement of potency (IC_50_ = 0.062 μM).

Interestingly, the replacement of the cyano group in C6 of structure **24** with an endocyclic nitrogen atom led to compound **25**, endowed with similar biochemical potency (IC_50_ = 0.052 μM) and better ligand efficiency (LE = 0.45), thus yielding an alternative core for SAR studies (scaffold II, Figure 11). Scaffold hopping investigations on rings A and B of **25** revealed that a certain positioning of nitrogen atoms on the core structure also played a relevant role. Whereas the removal of N1 (**26**) caused a 5-fold decrease of activity, when the nitrogen atom was removed from position 2 or 6 (**27**–**29**), the potency consistently decreased in the micromolar range as a result of a loss of hydrogen bond acceptors (see compound **25** in Figure 11 for the core numbering). Reduction of the number of heteroatoms as well as their mutual positions within the ring was also detrimental (**28** and **30**), but this can be ascribed to the decreased electron deficiency of the [1,2,4]triazolo[4,3-*a*]pyridine core, which is necessary to establish effective π–π contacts with Tyr148 and Tyr365 residues.^45^

**Figure 12:**
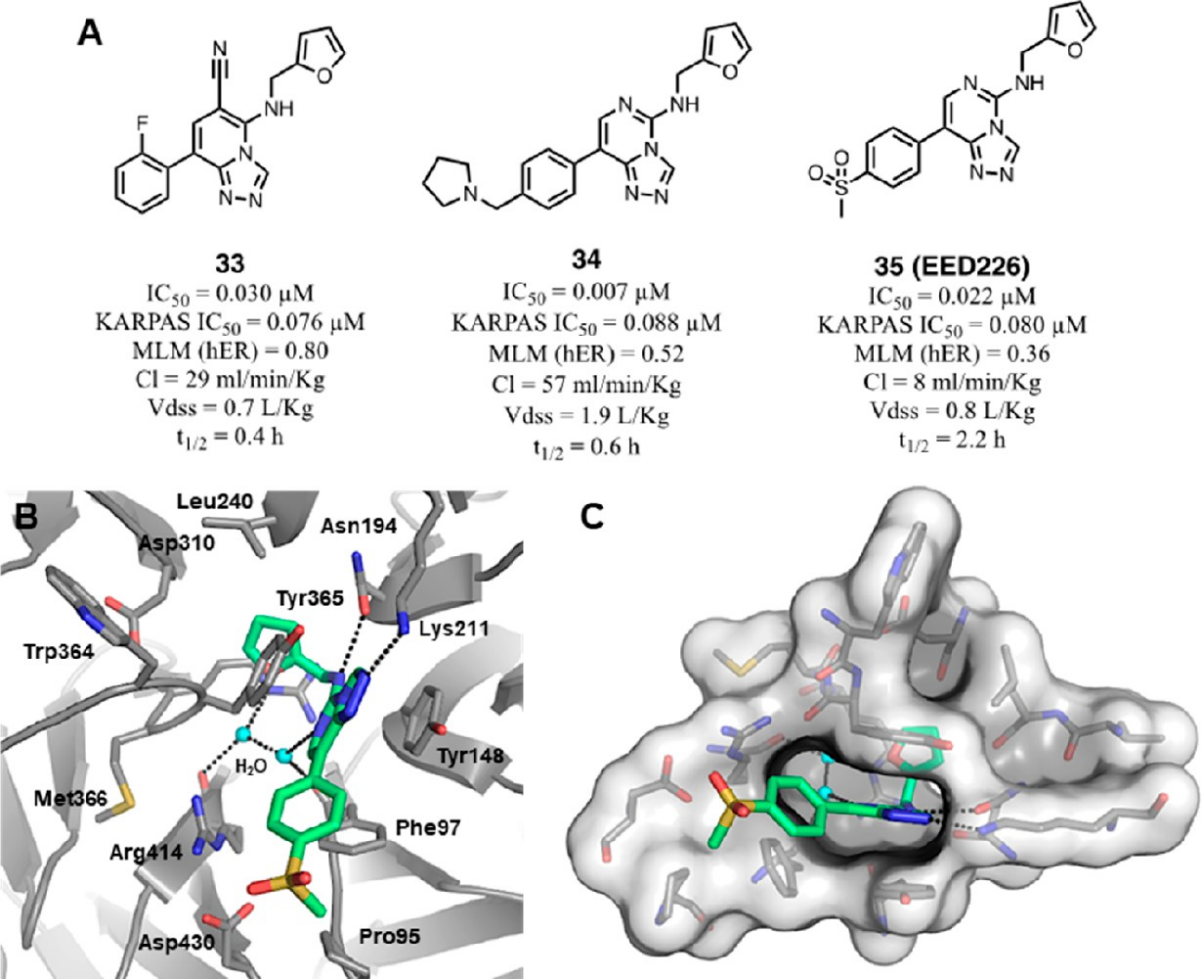
Main compounds obtained during SAR investigations on ring D and binding mode representation of EED–EBD–EED226 (**35**) ternary complex. (A) Structures of compounds **33**–**35** and their pharmacokinetic properties. (B) Binding mode of **35** (green) within the H3K27me3 pocket of EED (gray); interacting residues are labeled and represented as sticks, whereas water molecules are shown as cyan balls, and inter and intramolecular H-bonds are shown as dashed black lines (PDB 5GSA). (C) Binding mode of **35**, in green sticks, embedded in EED cavity shown in transparent white surface representation. Residues surrounding the binding pocket are represented in sticks, water molecules are represented as cyan balls, and inter- and intramolecular hydrogen bonds are represented as dashed black lines.

Huang et al. also reported that functionalization on the 3′- or 4′-position of the C8 aryl group on structure **24** with a dimethylamino group (**31a**) or other substituted amines (data not published) provided a further increase of the biochemical inhibition potency (**31a**, IC_50_ = 0.013 μM).^82^ The inclusion of the dimethylaminomethylene moiety on the previously identified [1,2,4]triazolo[4,3-*a*]pyridine and [1,2,4]triazolo[4,3-*c*]pyrimidine cores defined the scaffolds III and IV, which were used to carry out SAR investigations focusing on the “deep pocket” moiety (ring C, Figure 11). In this regard, different aromatic modules were evaluated, such as electron-rich (**31a/32a**, **31b/32b**, **31c/32c**) or electron-deficient five-membered rings (**31d/32d**, **31e/32e**) and structures with different steric hindrance including 3′-methyl- or 3′-chlorothiophenyl (**31f/32f**, **31g/32g**) and 6-membered phenyl or 2′-methoxyphenyl rings (**31h/32h**, **31i/32i**). Despite the good tolerance exhibited by electron-rich aromatic substitutions, these SAR studies identified the furan-2-yl ring as a critical element for potent PRC2 methyltransferase inhibition. Given the nanomolar activity of **31a** and **32a**, both showing an IC_50_ = 0.013 μM in a biochemical setting, the ensuing investigations focused on the solvent-exposed ring D functions to enhance the biological and PK profiles. The considered compounds (**33**–**35**, Figure 12A) showed nanomolar range activities, and among them, the 4′-methansulfonyl compound **35** (EED226) stood out as the best candidate for late *in vivo* studies (biochemical IC_50_ = 0.022 μM, *K*_D_ = 82 and 114 nM for EED and PRC2, respectively, in ITC evaluation). Notably, **35** exhibited complete oral bioavailability and a reasonable half-life (*t*_1/2_ = 2.2 h) due to its very low *in vitro* and *in vivo* hepatic clearance as predicted by the MLM (mouse liver microsome) assay.^82^

Qi et al. solved the crystal structure of EED in complex with **35** and the EBD peptide (PDB 5GSA) (Figure 12B), revealing that the compound retains all the fundamental interactions of the parent compound **12** and establishes “deep-pocket” interactions with the EED cavity by the furan-2-yl moiety.^83^ As for the cyano group in C6 of structure **12**, the endocyclic N6 nitrogen atom interacts with various residues (Phe97, Arg367, Arg414) via a water-mediated H-bond network. These water molecules fill the remaining volume in the deep pocket formerly occupied by the C6 cyano group and are essential to anchor **35** within the induced binding pocket (Figure 12C). The C8 (4′-methansulfonyl)phenyl moiety is mostly solvent exposed, but along its trajectory, its phenyl ring engages in an edge-to-face π–π interaction with the Phe97 side chain.^83^ In further biochemical evaluations, **35** was confirmed to compete with H3K27me3 for EED in AlphaScreen-based assessment and proved to be selective for the PRC2 complex over 67 different proteins including methyltransferases, kinases, GPCRs, ion channels, nuclear receptors, and transporters.^83^

After showing that **35** was able to induce a dose-dependent global decrease of both H3K27me3 and H3K27me2 levels in G401 tumor cells, the authors compared its cellular effects with those of EI1, a SAM-competitive EZH2 inhibitor. In particular, gene expression microarray studies were performed to evaluate the induction of expression pattern changes at a transcriptome level. In KARPAS422 cells, EI1 and **35** raised mRNA levels of the same genes in a dose-dependent manner, and these alterations were linked to a decrease of H3K27me3 deposition on promoter regions of corresponding genes.

The robust anticancer activity of **35** resulted in a dose- and time-dependent effect on the proliferation of KARPAS422 cells, a DLBCL cell line holding the Y641N EZH2 gain-of-function mutation. Subcutaneous administration of **35** in the mouse xenograft model of KARPAS422 demonstrated good tolerability with no apparent side effects. Moreover, **35** induced slower growth and reduction of the tumor volume after dosing for 21 days at different concentrations twice a day and complete tumor regression after dosage for 32-days at 40 mg/kg twice a day. Interestingly, in a SAM-dependent drug-resistant WSU-DLCL2 cell pool (W-R10) and clones (W-R10-#2, #5 and #22) containing Y111N and F120L EZH2 mutations, **35** inhibited the proliferation with a similar potency to that with the wild-type PRC2 complex; whereas in the same experiments, EI1 and the drug tazemetostat exhibited no cell growth inhibition. Finally, the combination of **35** and EI1 demonstrated synergistic antiproliferative effects, thus suggesting that coadministration of an EED binder and a SAM-competitive EZH2 inhibitor might provide more effective anticancer activity.^83^

Notably, **35** was also successfully converted into a bivalent chemical degrader able to efficiently and selectively degrade EED as much as EZH2 and SUZ12 proteins in a ubiquitin proteasome-dependent fashion.^84^ To achieve this goal, the authors developed PROTACs endowed with linkers of different lengths to ensure a functional proximity and orientation between the EED- and the VHL-binding modules. Among the synthesized PROTACs, the one able to simultaneously bind to EED and induce its degradation (UNC6852) was further biologically assessed and proven to lessen H3K27me3 levels and to hamper cell growth when tested in Pfeiffer and DB cell lines.^84^

### 5.3 Development of EEDi-5285

**Figure 13:**
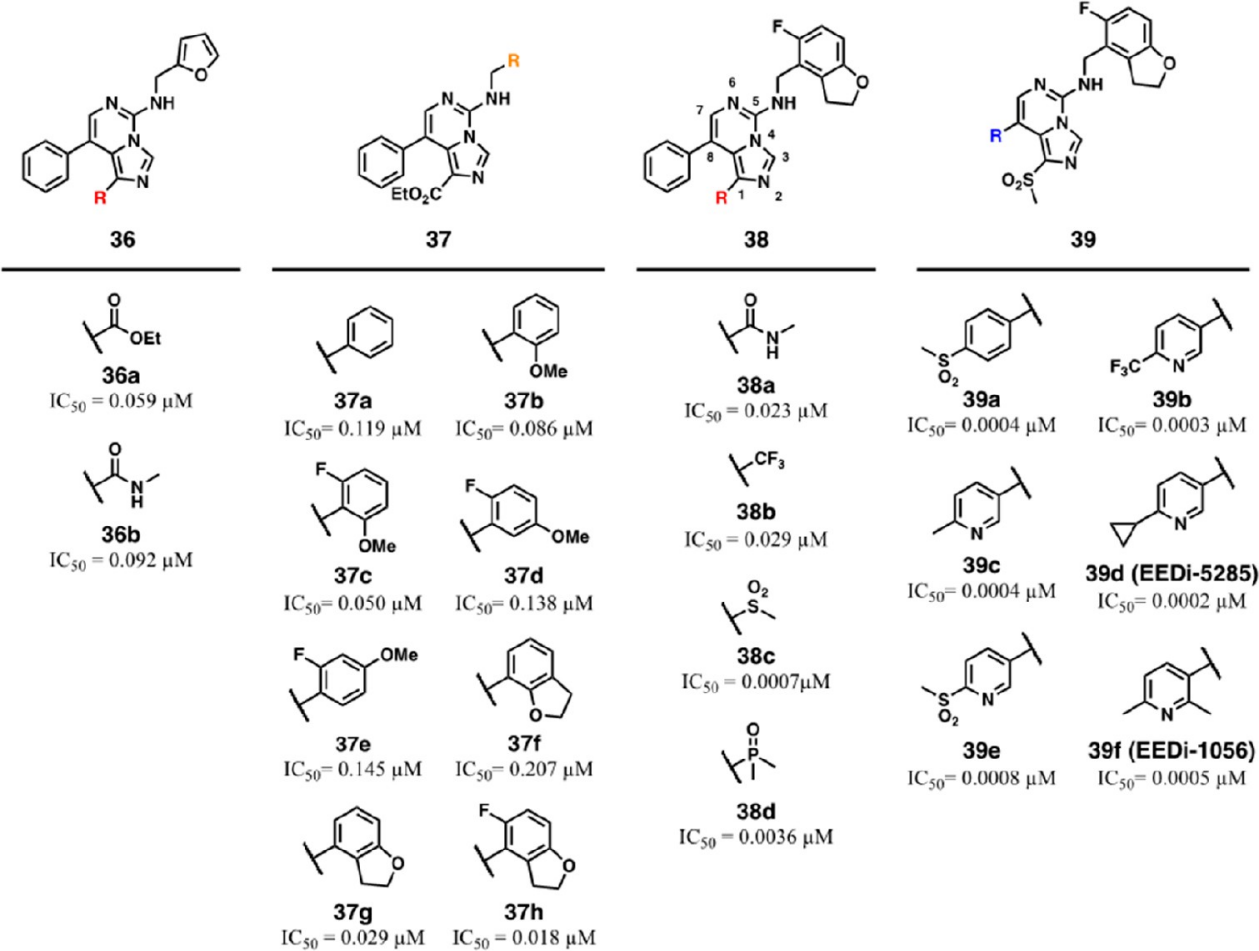
Panel of structural optimizations of compounds **36**–**39** and leading to EEDi-5285 (**39d**) and EEDi-1056 (**39f**).

A multidisciplinary team from the University of Michigan and Ascentage Pharma began a structure-guided discovery program of EED binders arising from a precursor of **35**, which in the end resulted in exceptionally potent, efficacious, and orally active compounds EEDi-5285 (**39d**) and EEDi-1056 (**39f**).^85^ Possessing a good antitumor activity in EZH2 mutant lymphoma models as well as in clones resistant to SAM-dependent EZH2 inhibitors, **35** represented a compelling core structure for further optimization. The structure-based campaign started from the previously reported inhibitor **26** (see Figure 11), which exhibited moderate competitive activity against EED–H3K27me3 binding in AlphaScreen assay (IC_50_ = 0.115 μM) and reasonable anticancer activity in KARPAS422 lymphoma cells (IC_50_ = 2.6 μM).^85^ Since the binding affinity to EED strongly depends on π–π stacking interactions with the electron-rich Tyr148/Tyr365 pair in the aromatic cage, the authors initially introduced small electron-withdrawing groups (EWGs; **36a**,**b**) on the imidazo[1,5-*c*]pyrimidine core of **26**. The methylamide-bearing **36b** was determined to have biochemical potency comparable to **26**, whereas the ethyl ester derivative **36a** was twice as potent (IC_50_ = 0.059 μM). Given the metabolic instability of the furan ring, further modifications concerned the moiety exploring the EED “deep pocket”. To reinforce the π–cation stacking interaction with the Arg367 guanidinium function at the bottom of the β-propeller cavity, an electron-rich 2-methoxy group was added to the phenyl ring (**37b**, Figure 13), and this compound displayed an IC_50_ value of 0.086 μM toward EED binding and IC_50_ of 1.9 μM against KARPAS422 cells, thus proving to be slightly more potent than the corresponding unsubstituted phenyl ring (**37a**). As a chemical probe, a fluorine atom was introduced at the C6 position of *ortho*-, *meta*-, and *para*-methoxy phenyl rings (**37c**–**e**). Although these modifications did not generally translate into a very strong improvement of activity in biochemical and cellular settings (KARPAS422), when a 2-fluoro-6-methoxyphenyl moiety was introduced (**37c**), a 1.7-fold increase of biochemical potency (IC_50_ = 0.050 μM) and an improvement of over 6-fold in cellular activity (KARPAS422 IC_50_ = 0.3 μM) was observed.

Further, the authors investigated the effects of phenyl ring replacement with electron-rich bicyclic systems (**37f**,**g**) by cycling the methoxy group with the phenyl ring on the deep binding pocket. Interestingly, the position of the oxygen in the dihydrobenzofuran nucleus had a significant impact on EED binding, with **37g** being 7-fold more potent than the corresponding regioisomer **37f** (IC_50_ = 0.029 μM and 0.207 μM, respectively). Moreover, the introduction of a fluorine atom at the C5 position of the 2,3-dihydrobenzofuran group (**37h**), described already in a previous Novartis patent,^78^ resulted in excellent IC_50_ values against EED (IC_50_ = 0.018 μM) and in cellular testing (IC_50_ = 0.012 μM), hence improving the antiproliferative activity in KARPAS422 more than 200-fold compared to **26**.

The encouraging results obtained with the substitution pattern of **37h** prompted the team to exhaustively probe the effect of EWGs in C1 of the imidazo[1,5-*c*]pyrimidine scaffold (**38a**–**d**). In this regard, *N*-methylcarboxamide, trifluoromethyl, methansulfonyl, and dimethylphosphine oxide were considered fruitful groups to strengthen the π–π interaction with the Tyr148/Tyr365 pair, and **38c** stood out as the most potent of the series in a biochemical setting (IC_50_ = 0.0007 μM).

Considering the predominant aromatic nature of **38c**, the next chemical manipulations were made around the C8 phenyl ring projecting outward from the EED cavity to improve the chemical–physical properties of the series. The effect of the aromatic ring in C8 was largely investigated by inserting suitable functional groups aiming either to increase the overall solubility of the compounds or to allow additional interactions over the external surface of EED (**39a**–**f**). In this regard, initial attempts led to compounds bearing a methansulfonyl group (**39a**), as in **35**, although this modification yielded limited binding improvement. Second, the phenyl extension was replaced with a variably decorated pyridine-3-yl ring to improve the solubility and simultaneously maintain favorable edge-to-face contacts with Phe97 (**39b**–**f**). Because the previously developed binder **35** established hydrophobic interactions with the external surface of EED, the pyridine-3-yl ring and the C8 position of the central scaffold (structures not shown, for further details see ref (85)) were decorated with mostly aliphatic moieties. Among these, the derivative endowed with a 4′-cyclopropyl group, **39d**, showed sub-nanomolar activity both on isolated EED (IC_50_ = 0.0002 μM) and in cell growth inhibition studies on KARPAS422 and Pfeiffer cells (IC_50_ = 0.0005 μM and 20 pM, respectively), thus being endorsed as the best candidate for further biological studies. Compound **39f** was also selected to be characterized by comparable biophysical affinity (IC_50_ = 0.0005 μM) and cellular antiproliferative activities (KARPAS422 IC_50_ = 0.0028 μM and Pfeiffer IC_50_ = 70 pM).

**Figure 14:**
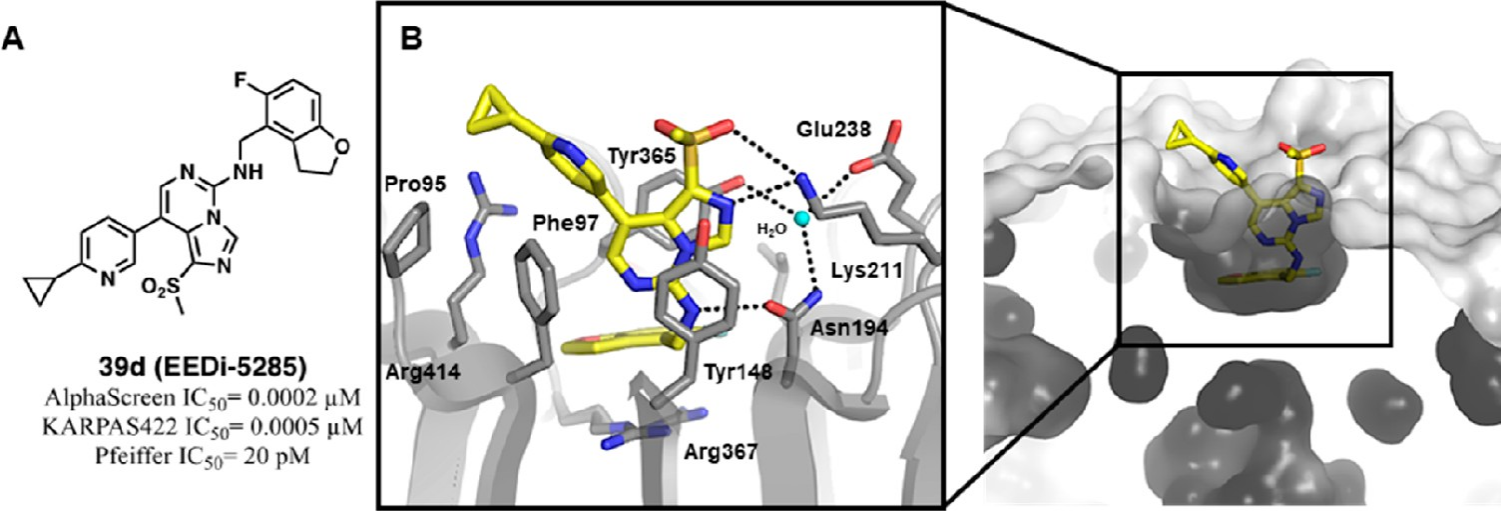
Chemical structure of EEDi-5285 (**39d**) and its binding mode representation within EED. (A) Chemical structure of EEDi-5285 (**39d**). (B) Cocrystal structure of compound EEDi-5285 (yellow) with the H3K27me3 pocket of EED (gray); interacting residues are labeled and represented as sticks, whereas water molecule is shown as a cyan ball, and intermolecular H-bonds are represented as dashed black lines (PDB 6W7F).

The binding mode of **39d** was determined via high-resolution cocrystal structure with EED (Figure 14), and comparison with the parent compound **35** rationalizes its extraordinarily potent interaction. Compound **39d** embeds within the H3K27me3 binding site through its 5-fluoro-2,3-dihydrobenzofuran group, which entirely fills the “deep pocket,” excluding the transient water molecules previously stabilizing the **35** structure (Figure 12B,C). Moreover, the 2,3-dihydrobenzofuran moiety establishes a stronger interaction with the Arg367 guanidinium group due to a better π–cation juxtaposition. At the entrance of the EED cavity, the methansulfonyl pendant in C1 provides a hot spot for H-bonding to the Lys211 side chain, thus strengthening the docking within the aromatic cage. Finally, the solvent exposed 3-pyridinyl engages in edge-to-face π–π interactions with Phe97 and orients its cyclopropyl moiety toward Pro95 where van-der-Waals interactions are positioned (Figure 14).

To evaluate the *in vivo* efficacy as antitumor agents, EEDi-5285 (**39d**) and the cognate compound EEDi-1056 (**39f**) were selected for explorative PK investigations. Both of them achieved acceptable plasma concentrations with oral bioavailability of 75% and 69%, respectively, and terminal *T*_1/2_ of approximately 2 h in a mouse model. In ensuing *in vivo* pharmacodynamic (PD) studies, **39d** and **39f** were evaluated in a KARPAS422 xenograft mouse model at doses of 50 and 100 mg/kg via daily oral administration. Notably, in a 28 day regimen, both of them induced complete tumor regression with minimal weight loss and were capable of a long-lasting activity since no tumor relapse was observed after 72 days by the end of the treatment.

### 5.4 Structural Optimization of Compound EED709

**Figure 15:**
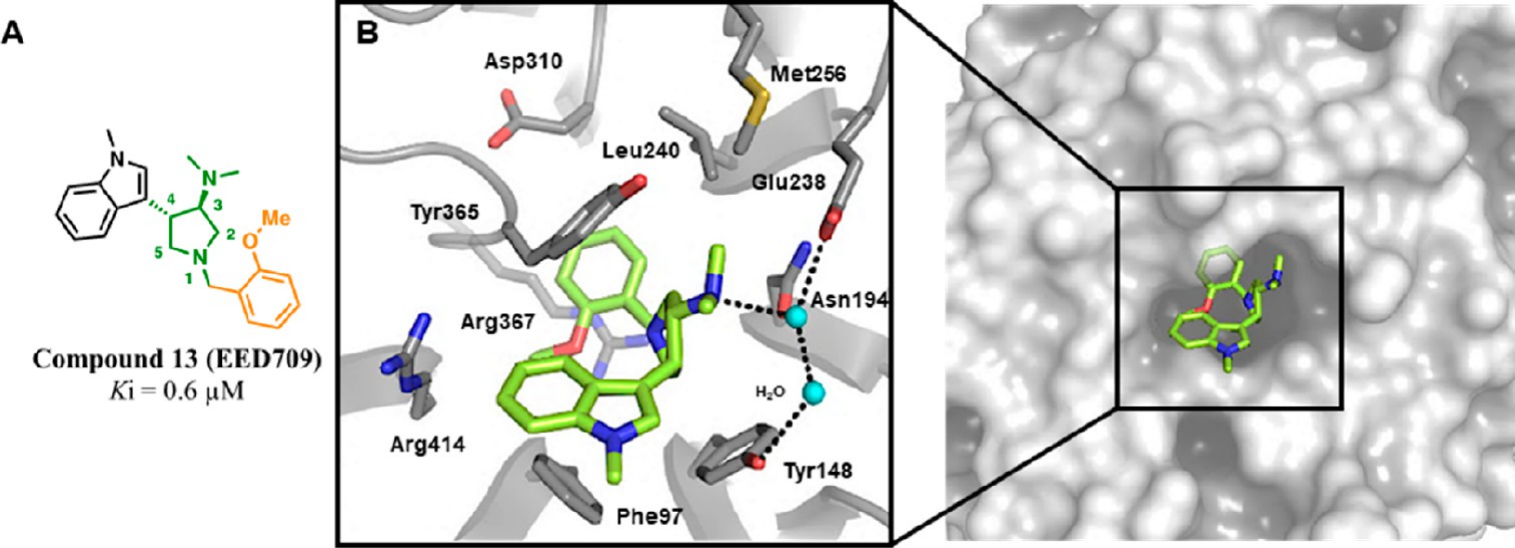
Chemical structure of **13** (EED709) and its binding mode representation within EED. (A) Chemical structure of compound **13** (EED709). (B) Cocrystal structure of compound **13** (light green) with the H3K27me3 pocket of EED (gray); interacting residues are labeled and represented as sticks, water molecules are shown as cyan balls, and intermolecular H-bonds are represented as dashed black lines (PDB 5U69).

When Novartis began the hit-discovery campaigns described above, AbbVie Inc. researchers similarly embarked on a high-throughput screening campaign to identify PRC2 allosteric inhibitors.^86^ Interestingly, employing a thermal shift assay (TSA)-based screening platform, they independently identified the same hit compound previously reported by Li et al.,^75^ EED709 (**13**, Figure 7A), as a sub-micromolar EED binder. This pyrrolidine-based hit was confirmed as an EED ligand by a time-resolved (TR)-FRET counter-screen, exhibiting a *K*_i_ of 0.6 μM, and its allosteric mechanism was ascertained on trimeric PRC2 *in vitro*. To shed light on its binding mode and guide chemical manipulations in a structure-based drug design approach, EED709 was crystallized with EED (Figure 15B). As seen for the other binders, the central cavity of EED undergoes significant remodelling upon ligand recognition, especially around Tyr365 and Arg367, and the newly formed space allows compound **13** to dock in the β-propeller structure. In this arrangement, the pyrrolidine core fits within the aromatic cage, directing the 2-methoxybenzyl moiety toward the Arg367, where it juxtaposes with the cationic guanidino group. The two pyrrolidine substituents in C3 and C4 protrude away from each other, the *N*,*N*-dimethylamino function contacts the Tyr148, Asn194, and Glu238 by a water network, and the *N*-methylindole flanks the rim of the EED pore where it engages in mostly hydrophobic interactions.

**Figure 16:**
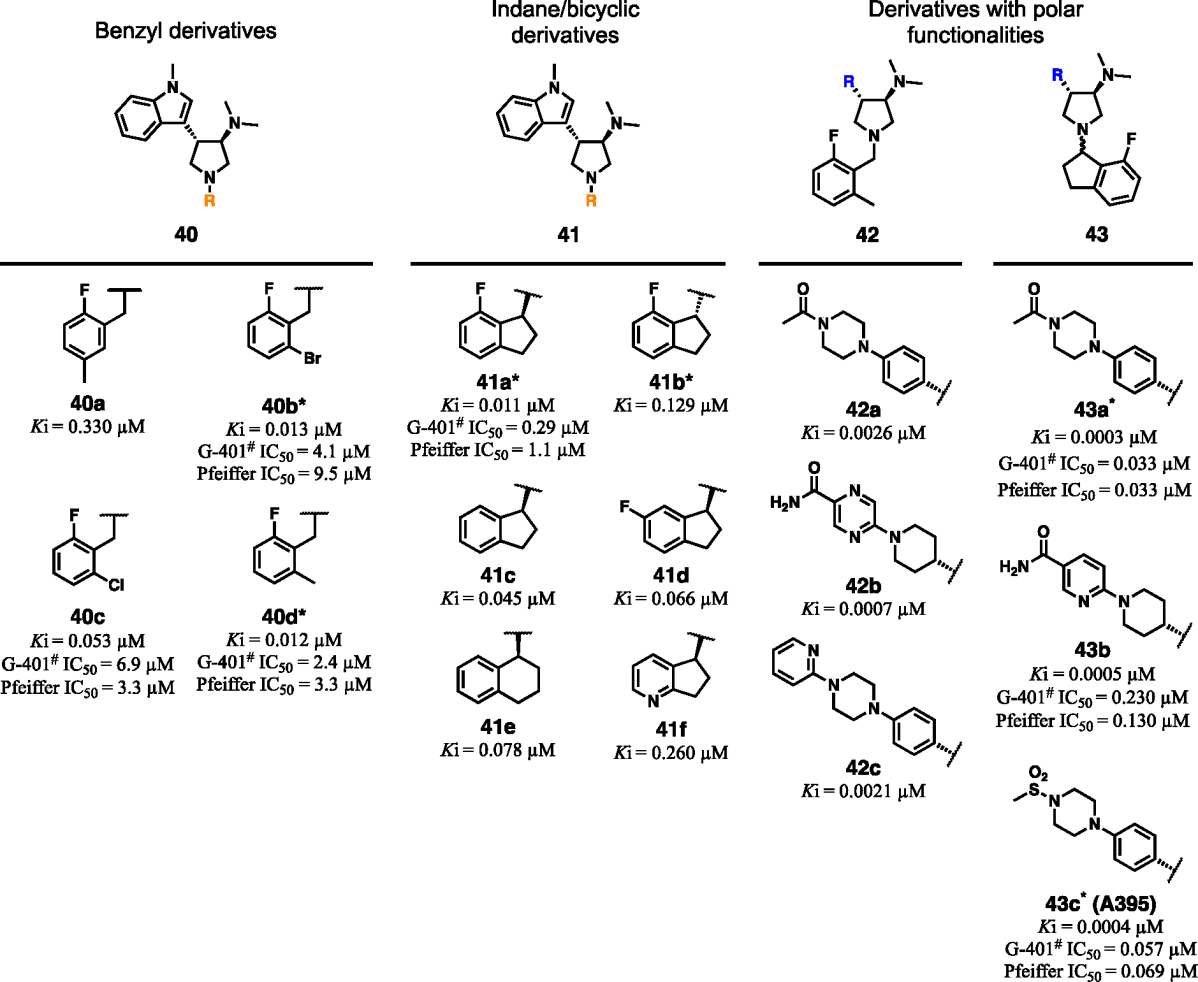
Structural modifications of compound **13** (EED709) leading to compounds **40**–**43**. All compounds are *trans-*racemic mixtures in C3–C4 unless otherwise noted with an asterisk. In the case of **43a** and **43c**, the *trans* racemic mixture was resolved, but the compound is an epimer at the indane stereocenter. ^#^IC_50_ values of H3K27 trimethylation inhibition over 6 days in tumor cells.

Starting from these insights, Curtin et al. began SAR studies along two lines of intervention (Figure 16): first, they probed the EED deep pocket with different mono- and bicyclic aromatic pendants (**40** and **41**); second, they investigated the solvent-exposed portion by replacing the *N*-methylindole with polar functionalities (**42** and **43**).^86^ The reported chemotypes presented two stereocenters at the C3 and C4 of the pyrrolidine (see Figure 15A for the core numbering) and, due to synthetic reasons, were obtained as a *trans*-racemic mixture. In addition to appraising the EED binding by TR-FRET assay, the most potent compounds were also tested in cancer cell lines responsive to EZH2 inhibition (i.e., G-401 and OCILY cells) to assess the impairment of the H3K27 trimethylation. Effects on proliferation in Pfeiffer cells, harboring EZH2 activating-mutations, were also evaluated. Initial efforts to enhance binding affinity revealed that replacing the 2-methoxyphenyl pendant with 2,6-disubstituted aromatic rings had a relevant impact on potency, leading to EED binders in the double digit nanomolar range (**40a**–**d**). The affinity improvement was related not only to electronic effects provided by the presence of halogens on the benzyl ring but more likely to the 2,6-substitution pattern, as observed comparing the 2-fluoro-5-methyl (**40a**) and 2-fluoro-6-methyl (**40d**) derivatives with the 2,6-dihalogenated **40b**,**c**. One step forward in this regard was represented by **41a** where the annulation between the *ortho* position and benzylic methylene led to a conformationally locked 7-fluoroindane analog with retained affinity in TR-FRET assay and increased inhibition of H3K27 trimethylation in kidney cancer cells (G-401 IC_50_ = 0.29 μM) and B cell lymphomas (OCILY IC_50_ = 0.66 μM). The indane moiety insertion induced an additional stereocenter; however, only the *S*-indane enantiomer showed low-nanomolar potency (**41a** being over 10-fold more potent than **41b**). In the attempt to reduce the lipophilicity of the series, SAR studies around the indane core and other bicyclic rings were also undertaken (**41c**–**f**). Nevertheless, despite the cLogP demonstrating better values (3.2 for **41f** versus a mean value of 4–5 for **40a**–**d** and **41a**,**b**), these modifications elicited a significant loss of affinity, up to10-fold, suggesting scarce space for improvement of the pharmacokinetic profile in this moiety.

Med-chem efforts on the solvent-exposed area led to better results in terms of balance between EED binding and lipophilicity. Indeed, projecting out of the H3K27me3 binding site, this moiety was more amenable to structural manipulations. So, placing polar extensions on the **40d** core structure (by replacing the *N*-methyindole scaffold) and using different *para*-substituted bicyclic linkers yielded single-digit to sub-nanomolar range ligands (**42a**–**c**) with reduced cLogP values (from 3.5 to −0.6). Finally, when some of the prior functionalities were combined with the 7-fluoroindan-1-yl pendant, the so-obtained EED binders exhibited sub-nanomolar potency in a TR-FRET assay and nanomolar cellular activities (**43a**–**c**). This series of compounds were obtained as *trans*-racemic mixtures, and only **43a** and **43c** were resolved as 3*R*,4*S* diastereomers, epimeric at the C1′ indane stereocenter.^86^ In particular, **43c** (A395) resulted in the most promising one and exhibited double digit nanomolar inhibitory activities either on H3K27 methylation in cancer G-401 and OCILY-19 cells or on Pfeiffer cell proliferation.

**Figure 17:**
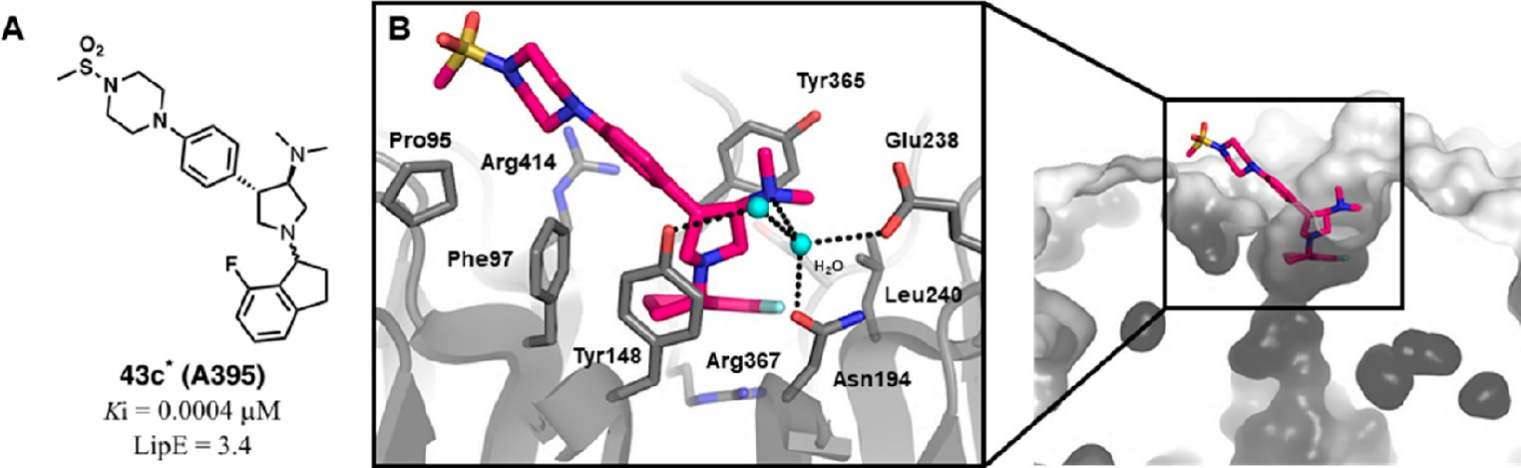
Chemical structure of **43c** (A395) and its binding mode representation within EED. (A) Chemical structure of compound **43c** (A395). (B) Binding mode of compound **43c** (A395) (magenta) within the H3K27me3 pocket of EED (gray); interacting residues are labeled and represented as sticks, water molecules are shown as cyan balls, and intermolecular H-bonds are represented as dashed black lines (PDB 5K0M).

Determination of its cocrystal structure with EED demonstrated that **43c** preserves the key interactions of EED709 and engages in enhanced π–cation interactions with Arg367 through its electron-rich 7-fluoro-indane cycle. Its *trans* configuration is determinant since it allows the dimethylamino moiety to take part in a water-mediated network of H-bonds with Tyr148, Asn194, and Glu238 while projecting the polar 4-methansulfonyl-piperazine extension out of the EED central pore toward a solvent-exposed area (Figure 17).^87^

High-throughput thermal shift assay against EED protein proved that **43c** markedly stabilized EED (Δ*T*_m_ = 13 °C at 50 μM), thus confirming its binding. In additional biophysical SPR experiments, **43c** bound to EED in 1:1 stoichiometry displaying a *K*_D_ value of 0.0015 μM. Moreover, evaluation of its inhibitory activity on the three-membered EZH2–EED–SUZ12 complex in a radioactivity-based assay revealed an IC_50_ of 0.0018 μM. When profiled against other epigenetic targets, **43c** proved to be selective for PRC2 up to 50 μM concentration over a panel of 32 different methyltransferases, including lysine, arginine, and DNA methyltransferases, and other epigenetic readers with trimethyl-lysine-recognizing capability. In cellular settings, **43c** blocked the H3K27 di- and trimethylation in rhabdoid tumor cells with IC_50_ values of 0.390 and 0.090 μM, respectively, and this inhibition was selective over other histone target methylation. Regarding *in vivo* studies, He et al. tested **43c** in a DLBCL Pfeiffer xenograft model by comparing its antitumor efficacy with the orthosteric EZH2 inhibitor GSK126, which due to PK issues, was administered subcutaneously at a dose of 300 mg/kg twice per week. In this experimental model, tumor growth inhibition (TGI) was reduced by 84%, while GSK126, administered at its established dosage protocol, elicited a TGI of 62%. Importantly, **43c** was less prone to induce resistance in Pfeiffer and KARPAS422 DLBCL cell lines and in drug-resistant clones retained antiproliferative activity,^87^ thus underscoring the potential of PRC2 allosteric inhibitors as an alternative therapeutic option to SAM-competitive EZH2 inhibitors.

## 6 Recent Advancements in EED Binder Development

**Figure 18:**
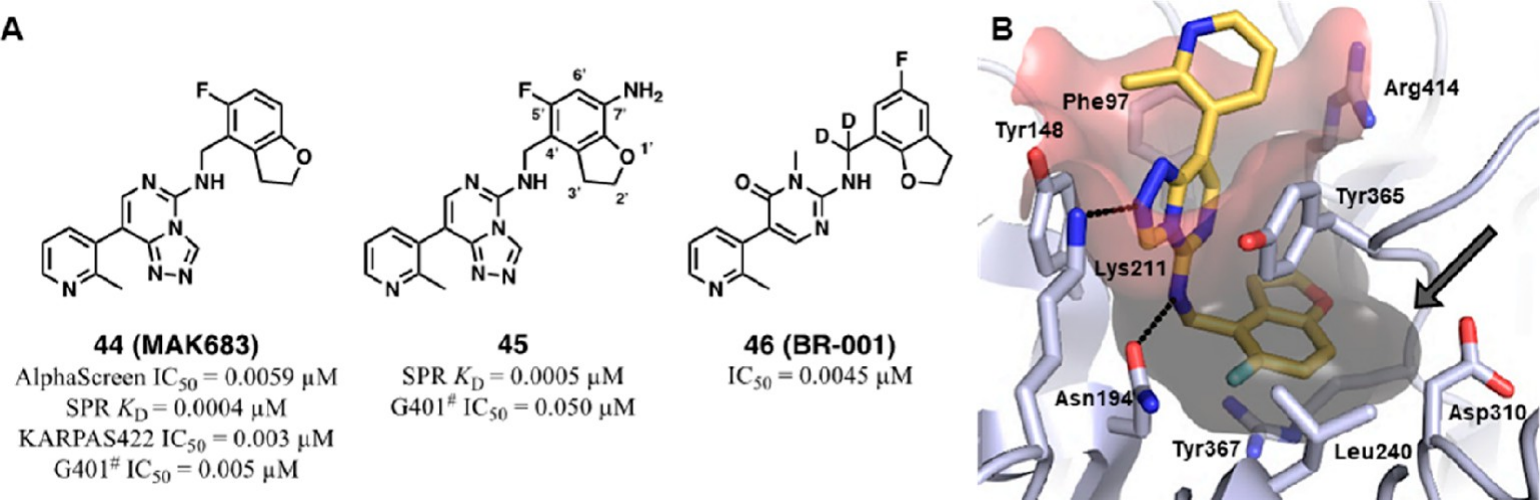
Chemical structures of **44**–**46** and binding mode of MAK683 within EED. (A) Chemical structures of **44** (MAK683), **45**, and **46** (BR-001). (B) Binding mode of compound **44** (MAK683) (gold) within the H3K27me3 pocket (in deep-salmon surface) of EED (blue-white cartoon); interacting residues are labeled and represented as sticks, intermolecular H-bonds are represented as dashed black lines (PDB 6YVJ). The dark arrow points to the EED surface facing the C7′ position of the dihydrobenzofuran ring of MAK683. ^#^IC_50_ values of H3K27 trimethylation inhibition over 6 days in tumor cells.

The above-discussed allosteric PRC2 inhibitors are excellent chemical tools proving the efficacy of tackling the EED H3K27me3-recognizing capability to cripple the PRC2 functions *in vitro* and *in vivo*. Following these efforts, Novartis developed another EED binder, MAK683 (**44**, Figure 18A), which is currently in phase I/II clinical trials for the treatment of DLBCL, nasopharyngeal carcinoma (NPC), or other advanced solid tumors (NCT02900651). MAK683 structure was reported in recent literature in Novartis’ patents together with other potent EED binders^78,88^ and by Huang et al.^89^ Similarly to EED226, it is characterized by a triazolo[4,3-*c*]pyrimidine core, but it features a 5-fluoro-2,3-dihydrobenzofuran ring, replacing the metabolically labile furan, which interestingly was shared in **39d** (EEDi-5285) as a fruitful “deep pocket” moiety. Compound **44** exhibited potent inhibition of EED–H3K27me3 peptide interaction in AlphaScreen experiments (IC_50_ = 0.0059 μM) and induced nanomolar antiproliferative effects in B cell lymphoma KARPAS422 cells (IC_50_ = 0.003 μM).^78^ Given its promising potential, very recently, **44** was employed to develop an EED-targeted PROTAC and drive the core PRC2 complex components to protein degradation, although with different kinetics.^90^ The solvent-exposed portion of **44** was exploited to functionalize the EED binder with the VHL warhead, and this structural modification proved to marginally alter the EED affinity and PRC2 inhibition *in vitro*. TR-FRET in vitro experiments revealed that EED–PROTAC–VHL ternary complex formation occurred only in the presence of PROTAC with the correct stereochemistry. Notably, the authors hypothesized that the diverse rate of protein degradation observed for EED, EZH2, and SUZ12 might imply different underlying mechanisms. On one side, EED is primarily targeted by the ubiquitin proteasome system; on the other, EZH2 and SUZ12 protein depletion might occur as a result of their instability outside the PRC2 complex or be induced by secondary ubiquitylation of a target lysine on EZH2 and SUZ12 upon EED–PROTAC–VHL ternary complex formation.^90^

Moreover, very recently, AstraZeneca researchers have launched a structure-guided optimization of **44** to enhance its unexpected poor solubility (5 μM at pH 7.4) and physicochemical properties.^91^ To achieve indications for structural optimizations, **44** was cocrystallized with EED (Figure 18B), and the 3D model was used as a platform for free energy perturbation (FEP) studies directed toward the C7′ position of the dihydrobenzofuran ring. The interaction surface facing C7′ presented an inward hollow (highlighted by a black arrow in Figure 18B) and was assumed to be amenable to insertion of small moieties (-F, -NH_2_, -OH, -CN) able to increase the compound solubility without dampening the binding affinity. A significant improvement of solubility (69 μM at pH 7.4) was obtained with **45** (Figure 18A), which retained excellent affinity in SPR experiments (*K*_D_ = 0.0005 μM) with respect to **44** (*K*_D_ = 0.0004 μM) but was 10-fold less efficient in inhibiting H3K27 trimethylation in G401 cells (**45** IC_50_ = 0.05 μM versus **44** IC_50_ = 0.005 μM).^91^

Starting from EED226, another potent and selective EED binder was recently developed by Zou and collaborators from Shanghai Blueray Biopharma.^54^ Compound **46** (BR-001) (Figure 18A) arose from a scaffold-hopping approach and featured a pyrimidone core structure bearing a 5-fluoro-2,3-dihydrobenzofuran-7-yl moiety at the C2 position (Figure 18A). Structural analysis on isolated EED uncovered a typical binding pose for **46**, with the 3-methylpyrimid-4-one scaffold lodging within the Phe97–Tyr148–Tyr365 aromatic cage where it engages in π–π stacking interactions. Protruding from this position, its benzofuran-7-yl fits within the induced-pocket of the β-propeller pore, whereas the 2-methylpyrimidin-3-yl projects outward to the solvent. In competition binding assays on EED, BR-001 displaced H3K27me3 with a low-nanomolar potency (IC_50_ = 0.0045 μM), and this activity translated well in a cellular setting (KARPAS422) where the compound dose-dependently inhibited H3K27 trimethylation and cell viability. To assess antiproliferative effects in a panel of cancer cell lines, **46** was tested in DLBCL cell lines, harboring EZH2-activating mutations, along with other cancer cell types characterized by EZH2 overexpression.

Interestingly, leukemia, gastric tumor, and brain tumor cells with altered levels of EZH2 did not respond to EZH2 inhibition by BR-001. Resistance to EZH2 inhibition was formerly linked to increased MLL1 expression level and H3K27ac upregulation. However, this effect was correlated to the induction of H3K27 acetylation rather than to MLL1 overexpression alone. *In vivo* KARPAS422 and Pfeiffer xenograft model studies demonstrated the ability of BR-001 to achieve 85% and 96% tumor growth inhibition, respectively, along with dose-dependent H3K27me3 level decrease and increased expression of PRC2-pertinent genes. Finally, the authors unveiled an unprecedented immunomodulatory mechanism of action for BR-001 and most likely for EZH2 allosteric inhibitors in general.^54^ Indeed, BR-001 resulted in robust upregulation of the CXCL10 chemokine levels in colorectal carcinoma cells that eventually led to CD8^+^ T cell recruitment to the tumor in murine models, which might expand the biological application of this antitumor agent.

## 7 Conclusions

In the last years, tremendous progress has been made in the development of small molecules directly or indirectly targeting EZH2. Recently, the EZH2 catalytic inhibitor tazemetostat hit the market after FDA approval for locally advanced epithelioid sarcoma and follicular lymphoma.^48−50^ However, the work for medicinal chemists is not finished yet as orthosteric EZH2 inhibitors induce resistance despite being already selective toward many other methyltransferases.

In 2014, after a structure-based virtual screening, the Luo group discovered astemizole (**1**) as the first-in-class compound able to disrupt the EZH2–EED interaction.^72^ Very recently, Luo’s group has provided the cocrystal structure of the EED–**1** complex clarifying its binding mode: **1** is able to bind to the bottom of the WD40-repeat domain of EED, thus hampering its binding to EZH2, but differently from EED inhibitors that bind to the H3K27me3 binding pocket at the top of WD40-repeat domain of EED.^72^ In the same work, a first SAR investigation on **1** revealed that a cyclic homology study led to a significant improvement in potency with compound **5b** that was the most potent (IC_50_ = 4.21 μM) among the novel analogues. Importantly, this new compound proved the ability to destabilize the PRC2 complex, leading to the degradation of EED, EZH2, and SUZ12 proteins,^72^ differently from the EED inhibitors, that contrarily stabilize the PRC2.^83,87^

The EED–**1** crystal structure highlighted that the EZH2–EED interaction interface could be targeted by small molecules and built the basis for future structure-based optimization and development of novel chemical entities able to display such a mechanism of action. In this regard, recent literature has already started to reveal novel structures able to disrupt the EZH2–EED PPI, and in the near future, we will observe the development of these early hits into optimized chemical tools.^92,93^ Given the extent of the EZH2–EED contact, we cannot exclude that among these chemical entities, there might be some able to dock outside or partially overlapping with the **1** binding site, thus broadening the molecular surface amenable for protein–protein disruption.

As seen above, Novartis published an interesting whole medicinal chemistry approach starting from a HTS campaign and identifying two hit compounds, **11** (EED210, IC_50_ = 2.5 μM) and **12** (EED162, IC_50_ = 4.03 μM). Both compounds underwent structural optimizations: particularly, the optimization campaign on **12** resulted in a very potent, selective compound, **35** (EED226, IC_50_ = 0.022 μM), with excellent *in vitro* and *in vivo* properties. EED226 impaired proliferation in EZH2 orthosteric inhibitor-resistant cancer cells and also achieved complete tumor regression in a mouse model. Of note, when **35** and the orthosteric EZH2 inhibitor EI1 were combined, synergistic antiproliferative effects were observed hence providing a more efficient anticancer strategy by combining two PRC2 modulators with two different mechanisms of action.

Very recently, from the University of Michigan and Ascentage Pharma, an optimization study on compound **35** led to an extremely potent and orally active compound, EEDi-5285 (**39d**, IC_50_ = 0.0002 μM). In *in vivo* studies, **39d** was evaluated through daily oral administration in a KARPAS422 xenograft mouse model, where it demonstrated complete tumor regression with minimal weight loss and also showed long-lasting activity since no tumor relapse was monitored after 72 days by the end of the treatment.

Concerning compound **11**, the optimization is still in its infancy, especially compared to the first series, as more med-chem optimization needs to be carried out before an evaluation *in vivo*.

Interestingly, AbbVie discovered almost in parallel via their high throughput screening the same hit **13** (EED709) that was identified by Novartis. The following structural optimization campaign led to compound **43c** (A395) with a *K*_i_ value of 0.0004 μM. When tested in a DLBCL Pfeiffer xenograft model, tumor growth was reduced by 84%. Notably, less resistance was observed during **43c** treatment of Pfeiffer and KARPAS422 DLBCL cell lines, therefore confirming that the PRC2 allosteric inhibitors (EED binders) could help to overcome the resistance induced by the EZH2 catalytic inhibitors in cancer therapy.

A point to raise is the following: not only the optimization of already discovered hit compounds (EZH2–EED interaction inhibitors and EED binders), but also the discovery of additional targetable structures of this complex will be a feasible way to improve PRC2 modulation. In these regards, a surface dip (Glu579 pocket) that senses the methylation status of H3K36 has been discovered as an additional allosteric mechanism not yet fully understood. The methylation status of H3K36 does not affect the binding affinity of PRC2 for H3K27 but the catalytic activity. In this regard, unmethylated H3K36 increases PRC2 catalytic turnover; contrarily, when H3K36 is methylated, it results in diminished turnover. This explains why H3K27 and H3K36 are methylated in a mutually exclusive manner. The existence of this Glu579 pocket could pave the way for further innovative allosteric EZH2 modulators.^4^ Moreover, it would be fascinating to evaluate whether targeting the third essential protein within the PRC2, SUZ12, could also offer a chance to modulate the methyltransferase activity of PRC2. In the years to come, after successful clinical validation and the approval of the first EZH2 catalytic inhibitor tazemetostat and the growing body of literature around the PRC2 complex, we can expect further advancements in the understanding of its complex biological functions as well as its direct or indirect modulation through small molecules.

Noteworthy, the emerging “targeted degradation” approach was also applied to PRC2. Jin and co-workers, who described the first EZH2 degraders, claimed that solely inhibiting EZH2 without decreasing protein level was insufficient to reduce breast cancer cell proliferation. Contrarily, the EZH2 degraders were able to completely suppress tumor growth in a triple-negative MDA-MB-468 breast cancer mouse model.^94^ The same approach was applied by AstraZeneca researchers who reported the first proteolysis targeting chimeras (PROTACs) directed to EED that were capable of leading its degradation.^90^

Interestingly, their EED–PROTAC model, in addition to degrading EED, was proven to likewise induce the degradation of EZH2 and SUZ12 proteins, similarly to the EZH2–EED interaction inhibitor **1** and compound **5b**, and to markedly arrest cell proliferation of KARPAS422 cells containing the gain of function Y641N EZH2 mutation.

These additional and very recent findings confirm that PRC2 can be nowadays considered a “hot target” in cancer chemotherapy research. Both EZH2 and EED degradation by EZH2–EED interaction impairment (**5b**) or by EZH2–EED–PROTAC offer a significant additional opportunity to block the PRC2 oncogenic activity, above all in the treatment of cancer types resistant to the EZH2 orthosteric inhibitors.

Moreover, it is worth mentioning that although PRC2 is considered a major complex in tumor therapy, its biological relevance in cancers different from lymphomas still remains to be elucidated. Furthermore, the synergistic effect observed in cellular settings upon the combination between the EZH2–EED interaction inhibitor **1** and the EZH2 inhibitor EPZ005687 but also between the EED binder **35** and EZH2 inhibitor EI1 prompt further *in vitro* and *in vivo* studies for in-depth antitumor property validation. Finally, it could be interesting to investigate the combination or to develop hybrid molecules acting as PRC2 orthosteric and allosteric inhibitors or degraders and on other epigenetic targets involved in the same or converging biological pathways of PRC2 such as HDAC or LSD1.^95^

## Abbreviations Used

AEBP2: adipocyte enhancer-binding protein
DLBCL: diffuse large B-cell lymphoma
EBD: EED binding domain
EED: embryonic ectoderm development
EPOP: elongin BC and polycomb repressive complex 2 associated protein
EZH2: enhancer of zeste homologue 2
FEP: free energy perturbation
FRET: fluorescence resonance energy transfer
GPCR: G protein-coupled receptor
H3K27: histone 3 lysine 37
HAC: heavy atom count
HDAC: histone deacetylase
HMTase: histone methyltransferase
HTRF: homogeneous time-resolved fluorescence
IGF-1R: insulin-like growth factor 1 receptor
ITC: isothermal titration calorimetry
JARID2: jumonji and AT-rich interaction domain containing 2
LE: ligand efficiency
LipE: lipophilic efficiency
LSD1: lysine-specific histone demethylase 1
MEK: mitogen-activated protein kinase kinase
MLM: mouse liver microsome
NPC: nasopharyngeal carcinoma
PALI: phylogeny and alignment of homologous protein structures
PcG: polycomp group
PCL1–3: polycomb-like proteins 1–3
PI3K: phosphoinositide-3- kinase
PPI: protein–protein interaction
PRC1/2: polycomb repressive complex 1/2
PROTAC: proteolysis targeting chimera
PTM: post-translational modification
RbAp46/48: retinoblastoma associated protein 46/48
RARa: RAR-related orphan receptor alpha
SAH-EZH2: stabilized α-helix of EZH2
SAL: SET activation loop
SAM: *S*-adenosyl-l-methionine
SBD: SANT1-binding domain
SET: Su(var.)3–9, enhancer-of-zeste and trithorax
SRM: stimulation-responsive motif
STD: saturation transfer difference
STAT3: signal transducer and activator of transcription 3
SPR: surface plasmon resonance
SUZ12: suppressor of zeste 12
TGI: tumor growth inhibition
TR: time-resolved
TSA: thermal shift assay
VHL: Von Hippel–Lindau
